# Neuroprotective and nephroprotective effects of *Ircinia* sponge in polycyclic aromatic hydrocarbons (PAHs) induced toxicity in animal model: a pharmacological and computational approach

**DOI:** 10.1007/s11356-023-27916-z

**Published:** 2023-06-15

**Authors:** Asmaa Nabil-Adam, Fadia S. Youssef, Mohamed L. Ashour, Mohamed A. Shreadah

**Affiliations:** 1 Marine Biotechnology and Natural Products Lab (MBNP), National Institute of Oceanography & Fisheries (NIOF), Alexandria, Egypt; 2 Department of Pharmacognosy, Faculty of Pharmacy, Ain-Shams University, Abbasia, 11566 Cairo Egypt; 3 Department of Pharmaceutical Sciences, Pharmacy Program, Batterjee Medical College, PO Box 6231, Jeddah, 21442 Saudi Arabia

**Keywords:** PAHs; *Ircinia* sponge, Neurotoxicity, Nephrotoxicity, Molecular docking, ADME, Drug discovery, Industrial development

## Abstract

The present study investigated the neuroprotective and nephroprotective effects of the sponge *Ircinia* sp. ethyl acetate extract (ISPE) against persistent aromatic pollutants in vitro and in vivo. Different exponential experimental assays were applied to this study. An in vitro study to investigate the potential therapeutic effect of ISPE using antioxidants (for example, ABTS and DPPH) and anti-Alzheimer assays (inhibition of acetylcholinesterase); the in-vivo study was designed to evaluate the protective effect of ISPE as neuroprotective and nephroprotective against the destructive effect of PAH. Several assays included oxidative assays (LPO), antioxidant biomarkers (GSH, GST), and inflammatory and neurodegenerative biomarkers (PTK,SAA). Additionally, the results were confirmed using histopathological examination. The in silico screening study improved the in vitro and in vivo findings through interaction between the aryl hydrocarbon receptor (AHR) and the polyphenolic content of ISPE extract, which was determined using LCMSM. The results and discussion showed that ISPE exhibited a promising antioxidant and anti-acetylcholinesterase activity as evidenced by IC_50_ values of 49.74, 28.25, and 0.18 µg/mL in DPPH, ABTS, and acetylcholinesterase inhibition assays, respectively. In vivo, the study showed that animals receiving ISPE before poly aromatic hydrocarbons administration PAHs (Prot, ISPE) showed significant amelioration in kidney functions manifested by the reduction of serum urea, uric acid, and creatinine by 40.6%, 66.4%, and 134.8%, respectively, concerning PAH-injected mice (HAA). Prot, ISPE revealed a decline in malondialdehyde (MDA) and total proteins (TP) in kidney and brain tissues by 73.63% and 50.21%, respectively, for MDA and 59.82% and 80.41%, respectively, for TP with respect to HAA. Prot, ISPE showed significant elevation in reduced glutathione (GSH) and glutathione transferase (GST) in kidney and brain tissues and reduction in the inflammatory and pre-cancerous biomarkers, namely, serum protein tyrosine kinases (PTKs) and serum amyloid A (SAA). These findings were further supported by histopathological examination of kidney and brain tissues, which revealed normal structure approaching normal control. Metabolic profiling of ISPE using LC–MS-MS showed the presence of fourteen polyphenolic compounds belonging mainly to phenolic acids and flavonoids. In silico study revealed that all the tested compounds exerted certain binding with the aryl hydrocarbon receptor, where rutin showed the best fitting (Δ*G* =  − 7.6 kcal/mol^−1^) with considerable pharmacokinetic and pharmacodynamic properties revealed from in silico ADME (Absorption, Distribution, Metabolism, and Excretion) study. Hence, it can be concluded that the *Ircinia* sponge showed a promising protective effect versus kidney and brain toxicity triggered by PAHs.

**Graphical Abstract:**

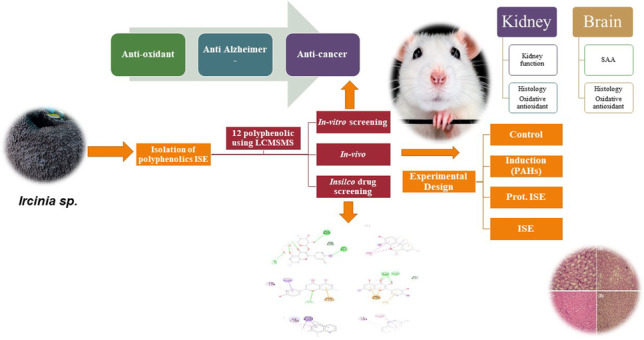

**Supplementary Information:**

The online version contains supplementary material available at 10.1007/s11356-023-27916-z.

## Introduction

During our daily life, humans are exposed to various environmentally hazardous agents from food, air, and water, in which hydrocarbons constitute the most dangerous compounds that consequently affect renal function with subsequent cumulative and progressive renal injury (Shriadah 1998). The great consumption of fossil fuels leads to increased environmental pollution, as hydrocarbons are mutagenic and carcinogenic substances with concomitant elevation in cancer risk (Boström et al. 2002). The great involvement of organic pollutants such as hydrocarbons in many industries has eventually led to the contamination of many aquatic resources (El Deeb et al. 2007). One of the most remarkable physiological effects is on various organs, particularly in fatty tissues (Abd El Moneam et al. 2016). PAHs produce various intermediates and end products after mixed-function oxidase metabolism via different metabolic pathways committed by the cytochrome P450 (Kumari et al. 2021). The metabolic products of PAHs covalently bind with crucial biological molecules within the human body comprising DNA forming complex adducts (PAH-DNA) that are consequently followed by mutation and DNA damage promoting carcinogenesis (Ugochukwu et al. 2018). Besides, hydrocarbon toxicity mainly targets the liver and kidneys in addition to triggering inflammation all over the body and central nervous system (CNS), consequently leading to mortality and morbidity duodenal disorders (Block and Calderón-Garcidueñas 2009; Kumar et al. 2021). Moreover, the pro-inflammatory signals caused by PAHs result in the massive production of ROS, cytokines, and particulate matter in the brain parenchyma, further contributing to CNS pathology. Meanwhile, chronic kidney disease (CKD) is a worldwide health burden providing a significant economic effect on health systems. It is an independent risk factor for cardiovascular disease (CVD), so it constitutes a serious challenge to global health as it increases among individuals of all ages (Carney 2020). The marine environment of the Red Sea was examined to combat PAH-related adverse effects, particularly those on the kidneys and the brain, using naturally occurring sources. This area is a significant source of different flora and fauna, including corals, bacteria, sponges, fungi, seaweeds, ascidians, and diatoms and their associated organisms (Youssef et al. 2019; El-Kashef et al. 2020a, 2020b). They possess novel bioactive chemical compounds that reveal highly valuable bioactivities to humans. Red Sea organisms can survive extreme temperatures, salinity, moisture, and wave action (Abdel-Halim et al. 2007). They can withstand land-based threats like urbanization and coastal development, recreation, industries, tourism, power plants, wastewater treatment facilities, quarrying activities, coastal mining, oil bunkering, and habitat modification like filling and converting wetlands (Raeder et al. 2008). Therefore, they represent validated starting points for drug discovery that can positively influence human ailments (Hegazy et al. 2016). Marine sponges belonging to the genus *Ircinia* (family *Irciniidae*) were revealed to be a rich source of bioactive metabolites showing interesting biological activities such as antibacterial, cytotoxic, and anti-inflammatory as well as analgesic activity (Issa et al. 2003; Fakhr et al. 2006; Chevallier et al. 2006). This is due to the richness of the members of *Ircinia* with steroids, macrolides, peptides, and fatty acids (Rashid et al. 2001). Bioactive metabolites previously detected in *Ircinia* species include furanosesquiterpenoids (Heidary Jamebozorgi et al. 2021), scalarane and cheilanthane sesterterpenoids (Buchanan et al. 2001; Lai et al. 2015), polyhalogenated peptides (Fernández et al. 2020), indole alkaloids (Shinonaga et al. 1994; Abdjul et al. 2015), and terpenoids and their derivatives (Su et al. 2011).

The main objective of the present study is to investigate the association of PAHs such as acenaphthylene, fluorene, phenanthrene, anthracene, pyrene, benz[a]anthracene, chrysene, benzo[b]fluoranthene, benzo[k]fluoranthene, benzo[a]pyrene, indeno first-ever evaluation of *Ircinia sp*. sponge extract’s protective effect against hydrocarbon-induced brain and kidney inflammation damage in male BLB/C mice. *Ircinia* sp. sponge extract was qualitatively and quantitatively analyzed using LC–MS-MS. In addition, a molecular docking study was performed on the different metabolites identified in *Ircinia* sp. sponge extract to ascertain its bioactive potential further.

## Material and methods

### Material

#### Sample collection

A sponge (*Ircinia* sp.) sample was collected from Hurghada at the Red Sea (latitude: 27°11′37.5″; longitude: 33°50′48.4″) in 2018. This was authenticated according to prior identification by Dr. Nicole De Voogd under the Voucher specimen of the authenticated sponge with code RMNH POR.8628 at the National Institute of Oceanography and Fisheries (NIOF). Sediment samples were collected from Lake Burullus (Latitude: 31° 30′ N; Longitude: 30° 50′ E) in the coastal part of the north-central Delta region in 2018 (Abdel Ghani and Shreadah 2021).

### Methods

#### Preparation of *Ircinia* sp. sponge extract (ISPE)

Five hundred grams of the sponge material was cut into small pieces, followed by diethylacetate extraction (3 × 1.5 L). The organic fractions were combined, and solvent removal was achieved at reduced pressure and temperature (35 °C), according to Abd El-Moneam et al. (2017), to give 2 g of semi-solid residue.

#### Metabolic profiling of ISPE using LC–MS-MS

The LC–ESI–MS/MS metabolic profiling of polyphenolic compounds in ethyl acetate extract of *Ircinia* sp. was carried out using the ExionLC AC system for separation and the SCIEX Triple Quad 5500 + MS/MS system with electrospray ionization sponge (ESI). Separation is carried out using a ZORBAX Eclipse Plus C18 Column (4.6100 mm, 1.8 m). LC grade formic acid in water (option A) and acetonitrile (option B). A mobile phase of 0% B, 60% B, and 2% B was used from 0 to 1 min. Injection rates of 3 l or 0.8 mL/min. The same MRM run employed positive and negative ionization modes for some polyphenols. For this experiment, we used the following parameters: 25 psi for the curtain gas, 4500 V for both the positive and negative modes of the IonSpray, 400 °C for the source temperature, 55 psi for the ion source gases 1 and 2, a declustering potential of 50, 25 J for the collision energy, and 10 J for the collision energy spread (Uddin et al. 2014).

#### Determination of total polyphenolics contents of ISPE

Total polyphenolic content in ISPE was determined using the method previously described by Taga et al. (1984). Meanwhile, the total flavonoid content was determined by a colorimetric method, as previously reported by Zhishen et al. (1999). The absorbance was measured immediately at *λ* = 510 nm.

#### Extraction of PAHs

PAHs were defined in the sediment sample gathered from Lake El Burullus, the most polluted region (Shreadah et al. 2014). Thirty grams of the sample was extracted for 8 h using 250 mL of *n-*hexane in Soxhlet, then re-extraction for an additional 8 h with 250 mL of dichloromethane (Shreadah et al. 2011). Combination and concentration of the extracts were performed at 35 °C using rotary evaporator. Consequently, a pure nitrogen gas stream was used to reduce the volume of the prepared sample to 1 mL. The prepared extract was shaken with mercury to remove sulfur. Similarly to the sample, 30 g of anhydrous sodium sulfate was extracted and used as the blank. The prepared sample (1 mL) was then applied at the top of a glass column which was prepared using the slurry packing of florisil (20 g) then, alumina (10 g), and finally, anhydrous sodium sulfate (1 g). The column was then eluted using *n-*hexane (70 mL) followed by 50 mL of the solvent system *n*-hexane: dichloromethane in the ratio of 7:3 for obtaining aliphatic (alkenes) and aromatic hydrocarbon (polycyclic aromatic hydrocarbons), respectively.

#### Identification of PAHs using GC–MS

Before putting the samples into a Thermo Trace GC UltraTM gas chromatograph system (Thermo Scientific, USA), they were concentrated with a gentle stream of clean nitrogen. PAHs were measured using a fused silica capillary column, Thermo TR-35 MS, with 35% phenyl polysil-phenylene-siloxane. The temperature went from 90 to 140 °C at a rate of 5 °C per minute and stayed there for 1 min. The temperature was then raised from 140 to 250 °C at a rate of 3 °C per minute and held there for 1 min. The temperature was then raised from 250 to 300 °C at a rate of 20 °C per minute and held there for 1 min. The temperature of the detector and injector was set to 310 °C and 280 °C, respectively.

#### In vitro estimation of ISPE for their biological activities

1. In vitro cytotoxic activity determination of ISPE

In vitro, cytotoxic activity ISPE was determined using the method previously described by Kosanić et al. (2015). Briefly, different concentrations were prepared of the samples to be tested by dissolving in DMSO, which was further diluted with the cell culture medium such that 1% of the total volume is the final DMSO concentration in all treatments comprising the control group. Cells that received no treatment were considered negative.

2. In vitro antioxidant activity determination of ISPE

In vitro, antioxidant ISPE was determined using two popular antioxidant assays widely used to screen the antioxidant activity of natural compounds using DPPH and ABTS radical scavenging activity assays (Mamadalieva et al. 2021). The DPPH radical scavenging capacity was determined using a method previously reported by Amarowicz et al. (2000) with slight modifications. Using ELISA, absorbance was determined at 490 nm, where % scavenging was determined in accordance with the following equation: % scavenging = [(A control − A sample)]/A control × 100. ABTS + free radical decolorization assay was performed using the previously reported procedure (Mamadalieva et al. 2021). The decrease in the absorbance % was determined at 734 nm, estimated for each point where percent inhibition (%) is used to determine the antioxidant potential of the examined samples. The following equation calculated it: Scavenging activity (%) = (*A*_*o*_ − *A*_*x*_)/*A*_*o*_ × 100; *A*_*x*_ and *A*_*o*_ represent the absorbance of samples with and without extract at *λ* = 734 nm, respectively.

3. In vitro, acetylcholinesterase inhibitory (AChI) activity determination of ISPE

Determination of AChI activity of ISPE was performed by Moyo et al. (2010).

#### In vivo estimation of the biological activities of ISPE

1. Animals and treatments

Thirty-two adult white male BALB/C mice weighing 18 to 25 g were obtained from the Institute of Theodor Bilharz animal house. Standard laboratory conditions were adopted for the maintenance of the animals in which the temperature was 33 ± 3 °C; the humidity was 20 ± 2%, normal light duration cycles, in addition to the utilization of standard rodent chow. After 1 week of acclimatization, the protective effects of ISPE against toxicity induced using aromatic hydrocarbon mixtures were studied. All methods in the current study were relevant according to guidelines and regulations and in compliance with the ARRIVE guidelines.

2. Determination of polyaromatic hydrocarbons dose used for toxicity induction

The protective effect of the sponge extract *(Ircinia* sp.) on the kidney and brain, as well as its antioxidant activity against toxicity induced by PAHs (465.2439 ng/mL) comprising a*cenaphthylene*, fluorene, phenanthrene, anthracene, pyrene, phenanthrene, anthracene, pyrene, benz[a]anthracene, chrysene, benzo[b]fluoranthene, benzo[k]fluoranthene, benzo[a] pyrene, indeno[1,2,3-cd]pyren, benzo(g,h,i) perylene, was evaluated in vivo following the method, described by Honda and Suzuki (2020).

3. Experimental design

Thirty-two adult white male BALB/C mice with weights ranging between 18 and 25 g were used. Mice were assigned into four groups, 8 mice in each group. In the first group, the animals were subcutaneously administered with saline solution for 1 week and considered as a negative control group, whereas in the second group, the induced toxicity group (HAA), the mice were subcutaneously injected with PAHs in a dose of 465.2439 ng/mL per kg body wt, which was dissolved in 0.5 mL corn oil for 1 week; however, in the third group, the animals were intraperitoneally administered with 5 mg/100 g/body/weight/day of *Ircini* sp. extract for 1 week in accordance to Enkhmaa et al. (2005) and Kumar et al. (2010) and thus served as a positive control group (ISPE). Meanwhile, the mice in the fourth group, the protected group (Prot. ISPE), were intraperitoneally administered 5 mg/100 g/body/weight/day of *Ircini* sp. extract for 1 week as a protection dose before the subcutaneous administration of 465.2439 ng/mL per kg.b.wt PAHs (dissolved in 0.5 mL corn oil) for 1 week. At the end of the experimental period (1 week), mice were anesthetized using isoflurane. Blood samples were gathered and maintained for 15 min at room temperature. The sera were separated via centrifugation at 3000 rpm for 20 min, then stored at − 20 °C until additional analyses were utilized. Kidney and brain tissues were weighed and purified from blood and other adhering material using cold isotonic saline. Crude kidney and brain homogenates were prepared via homogenizing 1 g of each of the kidney and brain tissue in 5 volumes of cold 0.1 M sodium phosphate buffer saline (PBS), pH 7.4 using mortar at 4 °C followed by the centrifugation of the homogenate at 7000 rpm for 20 min at 4 °C. The supernatant was then subdivided into three portions to determine MDA, GSH, and GST, which was then stored at − 20 °C until subsequent assessments.

4. Blood analysis for biochemical parameters determination

The Biuret reaction determined the total proteins, as Gornall et al. reported (1949). The absorbance was measured *versus* blank at *λ* = 540 nm, whereas the total creatinine was estimated by Henry (1964), while total uric acid was calculated using Barham and Trinder’s previously described method (1972), whereas total urea was assessed according to Patton and Crouch’s previously reported assay (1977).

5. Determination of antioxidant and oxidative stress biomarkers

Determination of lipid peroxidation (LPO) was performed *by* measuring malondialdehyde (MDA) levels by what was previously described by Rehman (1984), in which the absorbance was determined at *λ* = 535 nm. Additionally, the GSH was estimated following the method by Gornall et al. (1949) in which precipitation of 0.5 mL of the brain and kidney homogenates was performed using 2 mL of trichloroacetic acids (TCA) followed by measuring the absorbance of the formed yellow color *versus* the blank at *λ* = 412 nm. Furthermore, GST was determined following Habig et al.’s method (1974). GST substrate (*p*-nitrobenzyl chloride) reacts with GSH forming a conjugate product with a maximum absorbance at *λ* = 310 nm.

6. Assessment of inflammatory and cancer biomarkers

PTK was determined using a Universal Tyrosine Kinase Assay Kit (Takara, Tokyo, Japan), where phosphorylation was first started by adding 40 μL of serial dilutions of PTK control or samples into each well with a micropipette in duplicate. Exactly 10 μL of 40 mM of ATP-2Na solution was added into each well, mixed well, and then incubated at 37 °C for 30 min. Phosphorylation of tyrosine was started by adding an ATP solution. As a pro-inflammatory marker, serum amyloid A was determined by ELISA commercial kit (Invitrogen, Camarillo, Calif) (Liu et al. 2020).

7. Histopathological examination using the light microscope

Brain and kidney tissues were fixed in formalin, dehydrated in increasing grades of alcohol, and cleaned by immersing the tissues in xylene three times for 1 h, then impregnated in melted paraffin and wax and dried in an oven at 60 C for 1 h. The specimens were then embedded in paraffin, which solidified at room temperature. A rotatory microtome was used to cut 5-m-thick sections, which were then mounted on clean glass slides and stained with hematoxylin and eosin (H&E) to examine histopathological changes (Laulumaa et al. 2018).

#### In silico study

A computational study was performed to investigate the mechanism of action of polyphenolic compounds identified in the *Ircinia* sp. sponge using autodocking Vina experiment of natural products and polychlorinated hydrocarbon using aryl hydrocarbon receptor (PDB: ID 5V0L) downloaded from the protein data bank.

1. Preparation of the target protein

The protein structure of the aryl hydrocarbon receptor complex with co-crystalized citric acid (CIT) ligand (PDB ID: 5V0L) was retrieved from the protein data bank (https://www.rcsb.org/pdb/home/home.do) (Laulumaa et al. 2018).

2. Preparation of ligand

The 3D structures of the natural compounds identified in *Ircinia* sp. sponge by LC–MS-MS were obtained from ZINC and PubChem Databases and PAHs extracted from sediment (Goedtke et al. 2020).

3. Molecular docking

Auto Dock Vina 1.0, a docking experiment application, was used in the present investigation. Auto Dock Tools 1.5.6 was utilized to generate the grid box that would later pinpoint the protein’s active site within its pocket. The coordinates for the grid were set as follows: *x* = 30, *y* = 24, *z* = 30, *z* = 24, *x* center = 8.909, *y* center = 26.883, *z* center = 0.381 npts. All of the program’s other settings have been left at their defaults. *PyMol* and BIOVINA Discovery Studio Visualizer 4.0 pogroms displayed the docking results (Vieira and Sousa 2019).

4. Pharmacodynamics, drug-likeness, and medicinal chemistry of ligands

Different polyphenolics identified from the marine sponge were assessed for their drug-like properties by Lipinski’s rule of five; pharmacokinetics and medicinal chemistry were calculated by employing the Swiss ADME web tools.

### Statistical analyses

A triplicate experiment’s mean was expressed as a mean + standard deviation (SD). Duncan’s test was used to analyze statistical comparisons following ANOVA. Statistical significance was determined by a *p* value of 0.05. Graphs were created using the GraphPad Prism 5 software.

## Results

### Metabolic profiling of *Ircinia* sp. sponge ethyl acetate extract using LC/MS/MS

**Fig. 1:**
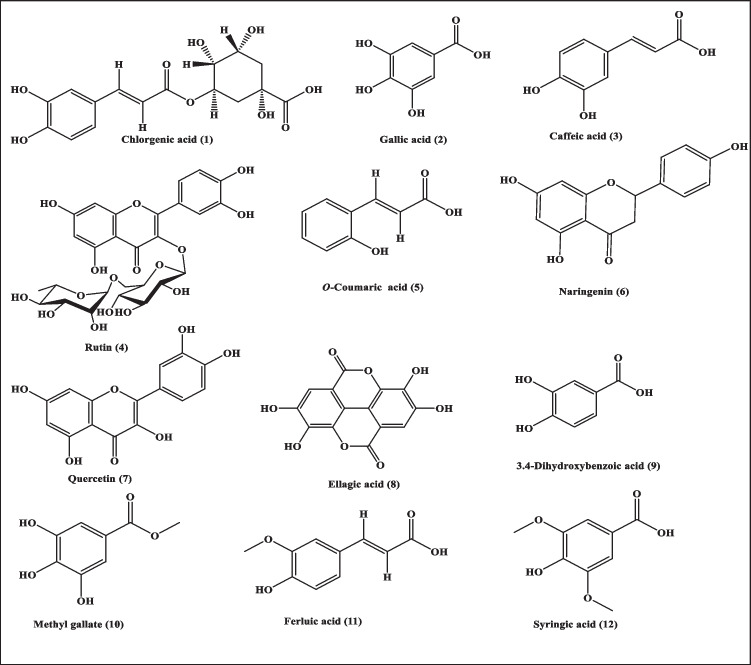
Scheme showing the Polyphenolic secondary metabolites identified in *Ircinia* sp. sponge ethyl acetate extract using LC/MS/MS

Metabolic profiling of *Ircinia* sp. sponge ethyl acetate extract (ISPE) using LC/MS/MS showed the presence of various polyphenolic compounds belonging mainly to phenolic acids as flavonoids. Twelve polyphenolic compounds were identified from the studied *Ircinia* sp. sponge ethyl acetate extract (ISPE), namely chlorogenic acid (1), gallic acid (2), caffeic acid (3), rutin (4), O-coumaric acid (5), naringenin (6), quercetin (7), ellagic acid (8), 3.4-dihydroxybenzoic acid (9), methyl gallate (10), ferulic acid (11), and syringic acid (12) (Fig. 1). Results illustrated in Table 1 revealed that the highest phenolic content was detected for chlorogenic and caffeic acid, with concentrations of 863.67 and 77.94 μg/g, respectively. Meanwhile, for flavonoids, the highest concentration was observed for naringenin, with a concentration of 57.66 μg/g; the LC/MS/MS chromatogram and a heat map showing the different distribution of *Ircinia* sp. sponge extract’s polyphenolic compounds are illustrated in Figure S1 and S2, respectively in the supplementary material*.*

**Table 1:** Different polyphenolic compounds in ISPE using LC/MS/MS

Detected compounds	STD area	RT (min)	Area	RT	ng/mL	µg/g	Mwt	MS/MS
Chlorogenic acid (1)	1,335,000	7.3	5,765,000	7.31	431.84	863.67	355.1	163
Gallic acid (2)	2,115,000	3.84	42,250	3.85	2.00	2.00	168.9	124.9
Caffeic acid (3)	9,708,000	7.99	3,783,000	8	38.97	77.94	178	135
Rutin (4)	15,740,000	9.69	1,507,000	9.69	9.57	19.15	609	299.9
Coumaric acid (5)	11,840,000	9.46	752,500	9.47	6.36	12.71	162.9	119
Naringenin (6)	40,860	14.99	11,780	14.9	28.83	57.66	271	119
Quercetin (7)	574,000	13.52	179,900	13.51	3.23	6.45	301	151
Ellagic acid (8)	243,700	9.89	10,060	9.89	4.13	8.26	301	145
3.4-Dihydroxybenzoic acid (9)	1,040,000	5.69	100,900	5.7	9.701	19.40	152.9	109
Methyl gallate (10)	14,100,000	7.39	50,620	7.39	0.36	0.72	183	124
Ferulic acid (11)	556,400	10.17	9367	10.17	1.68	3.37	192.8	133.9
Syringic acid (12)	224,600	8.34	41,680	8.35	18.56	37.11	196.8	181.9

### Determination of polyphenolics contents of ISPE

ISPE showed high phenolic and flavonoid contents, estimated at 7.218 and 3.093 mg/mL, respectively. In contrast, the tannic content showed a lower 0.018 mg/mL concentration than phenolic and flavonoid compounds. In addition, the total carbohydrates and sulfated polysaccharides concentrations were 0.849 and 0.1247 mg/mL, respectively they are listed in Table 2.

**Table 2:** The total phytochemical contents of the *Ircinia* sp. sponge extract

Bioactive compounds	Con. mg/mL
Phenolic	7.218
Flavonoids	3.093
Tannic	0.018
Sulfated polysaccharides	0.1247
Total carbohydrates	0.849

### Identification of PAHs extracted from Lake El Burullus sediments using GC–MS

The concentrations of different polycyclic aromatic hydrocarbons extracted from Lake El Burullus sediments are identified using GC–MS and illustrated in Table 3. Twelve PAHs were detected in Lake El Burullus sediments accounting for a total concentration of 448.66 ng/mL.

**Table 3:** Different PAHs extracted from Lake El Burullus sediments

	Polycyclic aromatic hydrocarbons (PAHs) name	Retention time		Concentration
		Standard	Sample	(ng/g)
1	Acenaphthylene	11.71	11.73	115.15
2	Fluorene	12.95	12.96	34.87
3	Phenanthrene	15.36	15.22	45.20
4	Anthrathene	15.42	15.33	50.69
5	Pyrene	19.36	19.27	54.61
6	Benzo(a)anthracene	22.85	22.84	7.13
7	Chrysene	23.08	23.10	9.73
8	Benzo(b)flouranthene	26.05	26.07	12.42
9	Benzo(K)flouranthene	26.13	26.15	9.72
10	Benzo(a)pyrene	27.23	27.24	34.61
11	Indeno(1,2,3_cd) pyrene	30.76	30.77	29.20
12	Dibenzo(a,h)anthracene	30.79	30.81	45.33

### In vitro antioxidant, cytotoxic, and anti-acetylcholinesterase activity

**Fig. 2:**
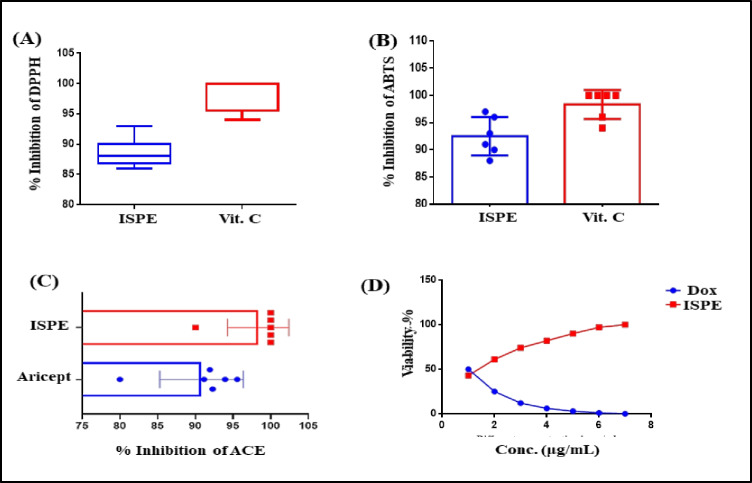
Total antioxidant capacity using DPPH (**A**), ABTS **(B)**, acetylcholine esterase inhibition **(C)**, and cytotoxic effect against HepG2 cell line **(D)** of ISPE

The total antioxidant capacity of ISPE was evaluated using DPPH and ABTS assays and compared with vitamin C. It exhibited promising antioxidant activity as evidenced by IC_50_ values of 49.74 and 28.25 μg/mL in DPPH and ABTS assays, respectively. However, vitamin C, the standard antioxidant drug, revealed IC_50_ of 5.53 and 5.61 μg/mL in DPPH and ABTS assays, respectively. It is worth highlighting that ISPE showed 86.73% and 88.17% inhibition of free radicals in DPPH and ABTS assays, respectively, at 1 mg/mL concentration. In addition, the inhibitory activity *versus* hepatocellular carcinoma cells was evaluated for ISPE that showed IC_50_ = 41.2 µg/mL. Meanwhile, ISPE exerted a pronounced anti-Alzheimer potential highlighted by acetylcholinesterase inhibition potential where ISPE showed a good inhibition potential with an IC_50_ value of 0.18 mg/mL showing 88.17% inhibition at 1 mg/mL. It approaches that of Aricept, the standard drug, with an IC50 value of 1.4198 mg/mL and a 90.03% inhibition at 1 mg/mL (Fig. 2).

### In vivo estimation of the biological activities of ISPE

1. Blood analysis for biochemical parameters showing kidney functions

Blood analysis results for biochemical parameters showing kidney functions are represented in Table 4. Administration of polycyclic aromatic hydrocarbons, HAA, significantly elevated serum urea, uric acid, and creatinine levels by 129.99%, 288.36%, and 184.85%, respectively, when compared to the normal control group. On the contrary, urea, uric acid, and creatinine serum levels were considerably reduced by 40.6%, 66.4%, and 134.8% in Prot. ISPE, respectively, with respect to polycyclic aromatic hydrocarbons, HAA-treated group. Thus, the protective effect of ISPE on kidney function was highly significant as it showed values approaching that of normal levels.

2. The pre-cancerous and inflammatory biomarkers

**Table 4:** Serum levels of urea, uric acid, and creatinine manifesting kidney function (mg/dL) in animals treated with ISPE after toxicity induction using PAHs

Name	Serum urea(mg/dL)	Serum uric acid(mg/dL)	Serum creatinine(mg/dL)
Control group	19.64 ± 3.38*	2.96 ± 0.37*	1.50 ± 0.15*
HAA group	45.17 ± 1.94	11.48 ± 0.82	4.27 ± 0.25
ISPE group	26.93 ± 5.30*	5.35 ± 0.35*	2.54 ± 0.18*
Prot. ISPE group	26.83 ± 6.16*	3.86 ± 0.38*	1.82 ± 0.17*

Results are expressed as mean ± SD

*HAA* polycyclic aromatic hydrocarbons treated group; *ISPE Ircinia* sp. sponge ethyl acetate extract treated group; *Prot. ISPE Ircinia* sp. sponge ethyl acetate extract–treated group followed by toxicity induction by polycyclic aromatic hydrocarbon-treated group

^*^p ≤ 0.001 compared to HAA-treated group

**Fig. 3:**
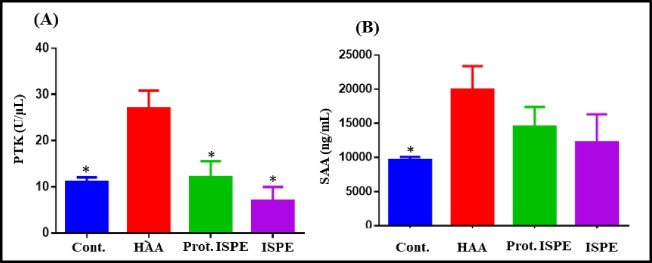
Serum levels of PTK (**A**) and SAA (**B**) in different experimental groups treated with *Ircinia* sp. sponge ethyl acetate extract after toxicity induction using polycyclic aromatic hydrocarbons. Results are expressed as mean ± SD, where *p ≤ 0.001 as compared to the HAA-treated group

Serum protein tyrosine kinases (PTKs) in blood were elevated greatly in a significant manner (*p* > 0.001, 27.58 ± 3.83) in the induction group (HAA), showing 59.83% elevation when compared to the control group (11.08 ± 1.04). In contrast, the protection group (Prot. ISPE) exhibited a significant decrease (*p* < 0.001*, 12.10 ± 3.39) in the PTK level, estimated by 56.13% when compared to the induction group. In the meantime, it showed a non-remarkable elevation compared to the control group (Fig. 3A). A similar attitude was observed in serum amyloid A (SAA) as it was significantly increased in the induction group, HAA, by 106.82% with regard to the control group. In contrast, the SAA level was significantly decreased (*p* = 0.002*, 14,501.89 ± 2927.99) in the protection group, Prot, ISPE, by 27.11% compared to the HAA group (19,897.39 ± 3493.32) (Fig. 3B).

3. The oxidative stress and antioxidant biomarkers in kidney and brain tissue

**Fig. 4:**
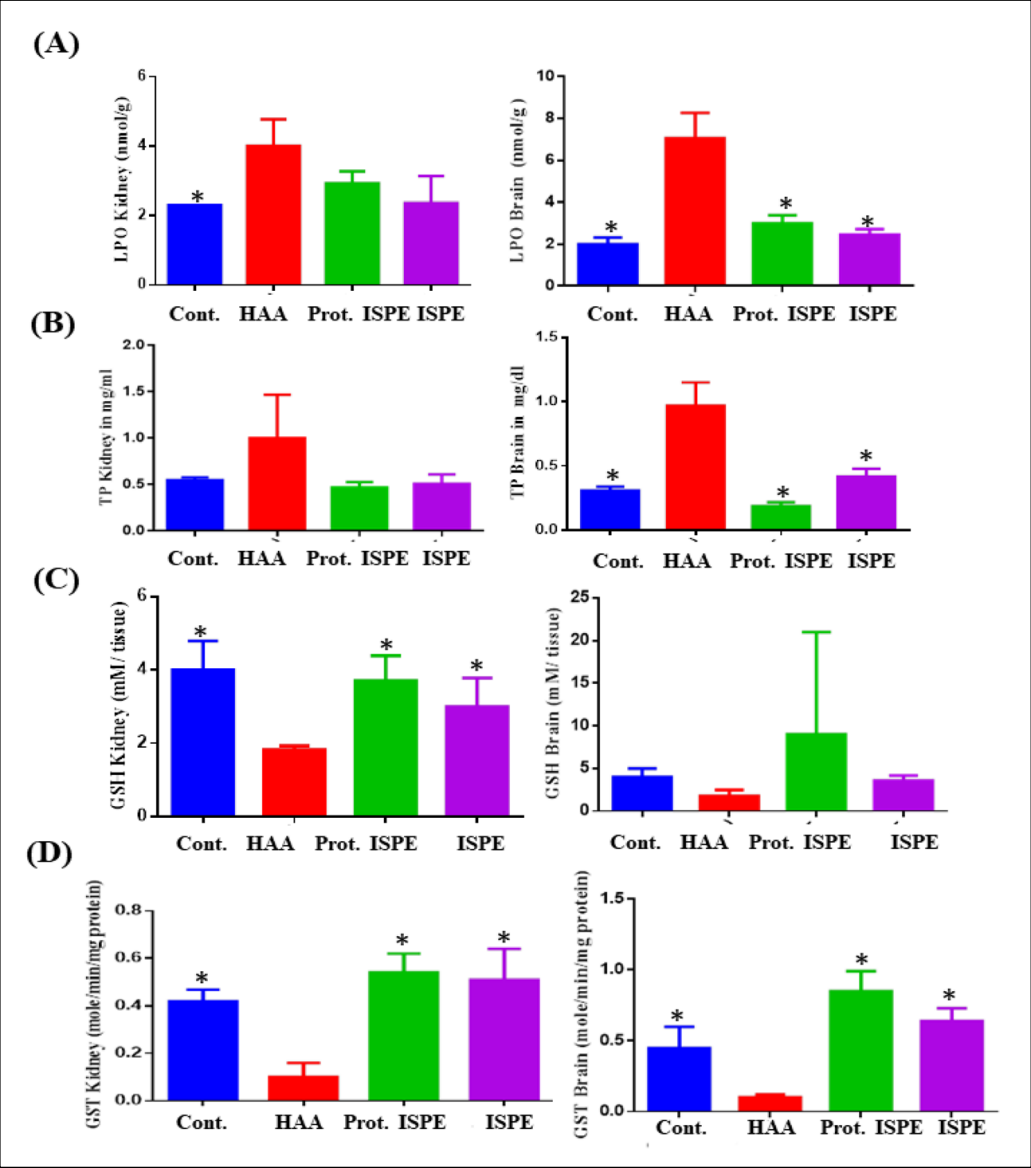
LPO (**A**), total proteins (**B**), GSH (**C**), and GST (**D**) in kidney and brain tissues in different experimental groups treated with *Ircinia* sp. sponge ethyl acetate extract after induction of toxicity using polycyclic aromatic hydrocarbons. Results are expressed as mean ± S.D, where *p ≤ 0.001 as compared to HAA-treated group

The malondialdehyde (MDA) level was measured in kidney and brain tissues to assess oxidative stress. The present study revealed that the induction group showed a great increase in the level of MDA in both kidney and brain tissues (*p* < 0.001*, 4.96 ± 0.77 and *p* < 0.001*, 7.05 ± 1.22) estimated by 53.63% and 172.22%, respectively with regard to the control group. In contrast, the protection group showed a lower level of MDA in both the kidney and brain (*p* < 0.001*, 2.92 ± 0.62 and *p* < 0.001*, 3.51 ± 0.39) estimated by 73.63% and 50.21%, respectively compared to the induction group with a non-significant increase than the control group (Fig. 4A). Similarly, with regard to total proteins (TP), the induction group showed a great increase in the level of TP in both kidney and brain tissues (*p* < 0.001*, 1.17 ± 0.03 and *p* < 0.001*, 0.97 ± 0.18) estimated by 112.7% and 212.9%, respectively, with regard to the control group (0.55 ± 0.03 and 0.31 ± 0.03). In contrast, the protection group showed a lower level of TP in both the kidney and brain (*p* < 0.001*, 0.55 ± 0.03 and *p* < 0.001*, 0.19 ± 0.03) estimated by 59.82% and 80.41%, respectively (Fig. 4B). Furthermore, the antioxidant biomarkers measured in the current study indicated that the levels of GSH were found to decrease significantly in kidney and brain tissues, induction group (*p* < 0.001*, 1.83 ± 0.53 and *p* < 0.001*, 1.75 ± 0.73), HAA, accounting for 60.81% and 64.14% reduction, respectively, with respect to the control group. Meanwhile, the protection group, Prot. ISPE, showed a significant elevation in GSH in kidney and brain tissues by 45.04 and 81.26%, respectively, with respect to the HAA group (Fig. 4C). Additionally, the levels of GST significantly reduced in kidney and brain tissues in the induction group (*p* < 0.001*, 0.10 ± 0.06 and *p* < 0.001*, 0.10 ± 0.02), HAA, accounting for 76.19% and 77.78%, respectively, with respect to the control group (0.42 ± 0.05 and 0.45 ± 0.15). Furthermore, the protection group, Prot. ISPE, showed a considerable elevation in GST in kidney and brain tissues (*p* < 0.001*, 0.54 ± 0.08 and *p* < 0.001*, 0.85 ± 0.14) by 81.48 and 88.23%, respectively, with respect to the HAA group (Fig. 4D).

4. Histopathological studies of kidney tissues

**Fig. 5:**
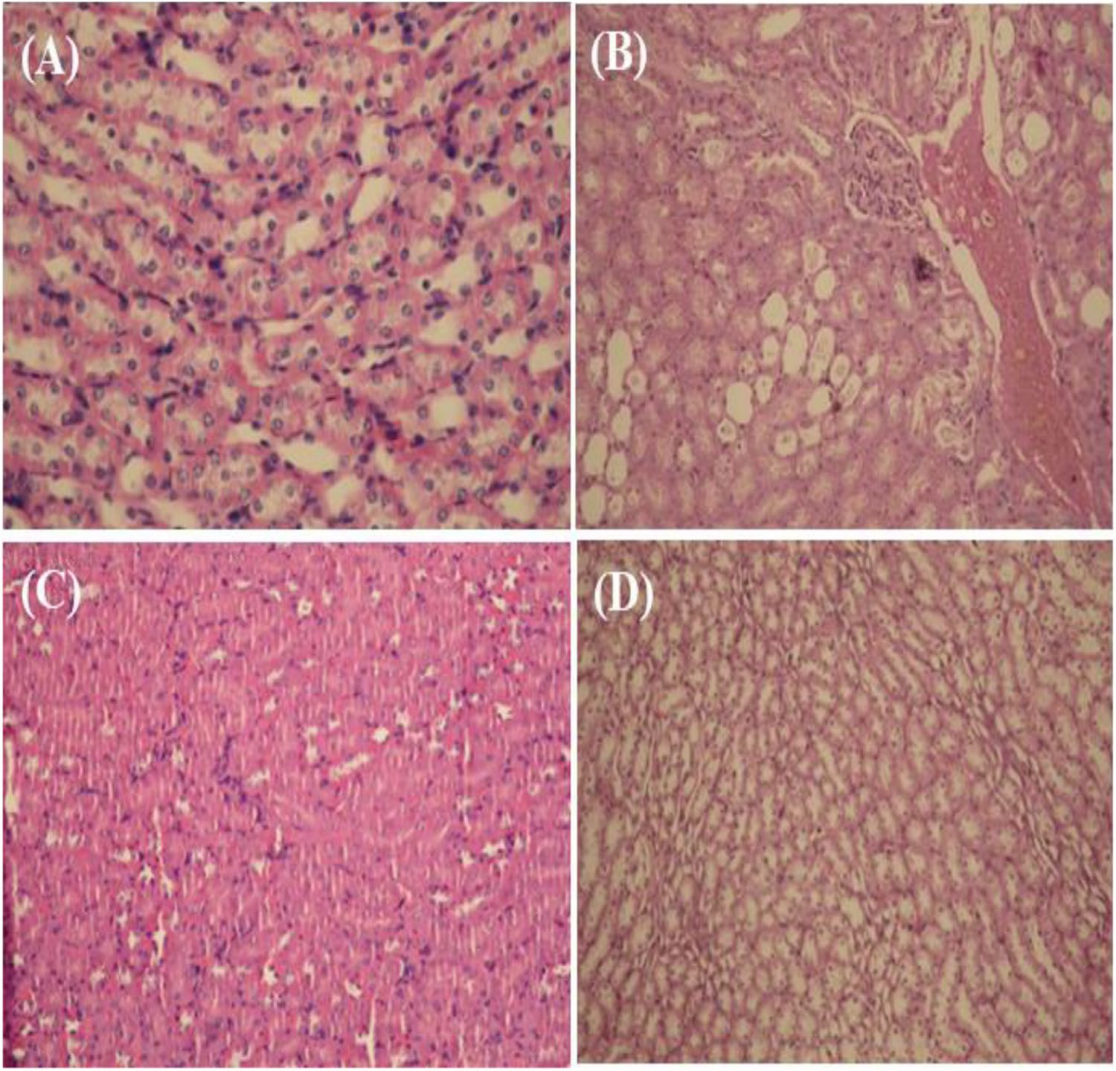
Histopathological examination of kidney tissues in **A** control group; showed normal renal architecture **B** HAA; increased obliterations in the urinary space among Bowman’s capsule glomerulus, blood vessel dilation and fibrosis; **C** ISPE showed normal renal architecture; and **D** Prot. ISPE showed significant decreases in kidney damage with great improvement in kidney structure

The histopathological results of kidney sections are illustrated in Fig. 5. The first group, the control group, showed normal renal architecture manifested by the normal structures of glomeruli, Bowman’s capsule, lined with squamous epithelium. Additionally, the urinary space was distinct, as revealed by the H&E stain × 400 (Fig. 5). In contrast to the control group, the induction group, treated with a hydrocarbon mixture, HAA, showed increased obliterations in the urinary space among Bowman’s capsule glomerulus. Other signs of kidney damage were seen in the kidney section of the induction group, such as hypertrophy and vascular congestion. It was also noted that the glomerulus was degenerating, the tubules were degenerating, eosinophilic casts were present within the tubule lumen, and blood vessel dilation and fibrosis were present. However, the protection group treated with the sponge extract before induction with hydrocarbon mixtures, Prot. ISPE, showed significant decreases in kidney damage with great improvement in kidney structure. Photomicrograph of kidney segment stained with H&E stain at 400 × reveals typical glomeruli, Bowman’s capsule, a clear urine space, proximal convoluted tubule lined with cuboidal epithelium, and distal convoluted tubule in the ISPE-treated group (Fig. 5).

5. Histopathological studies of brain tissues

**Fig. 6:**
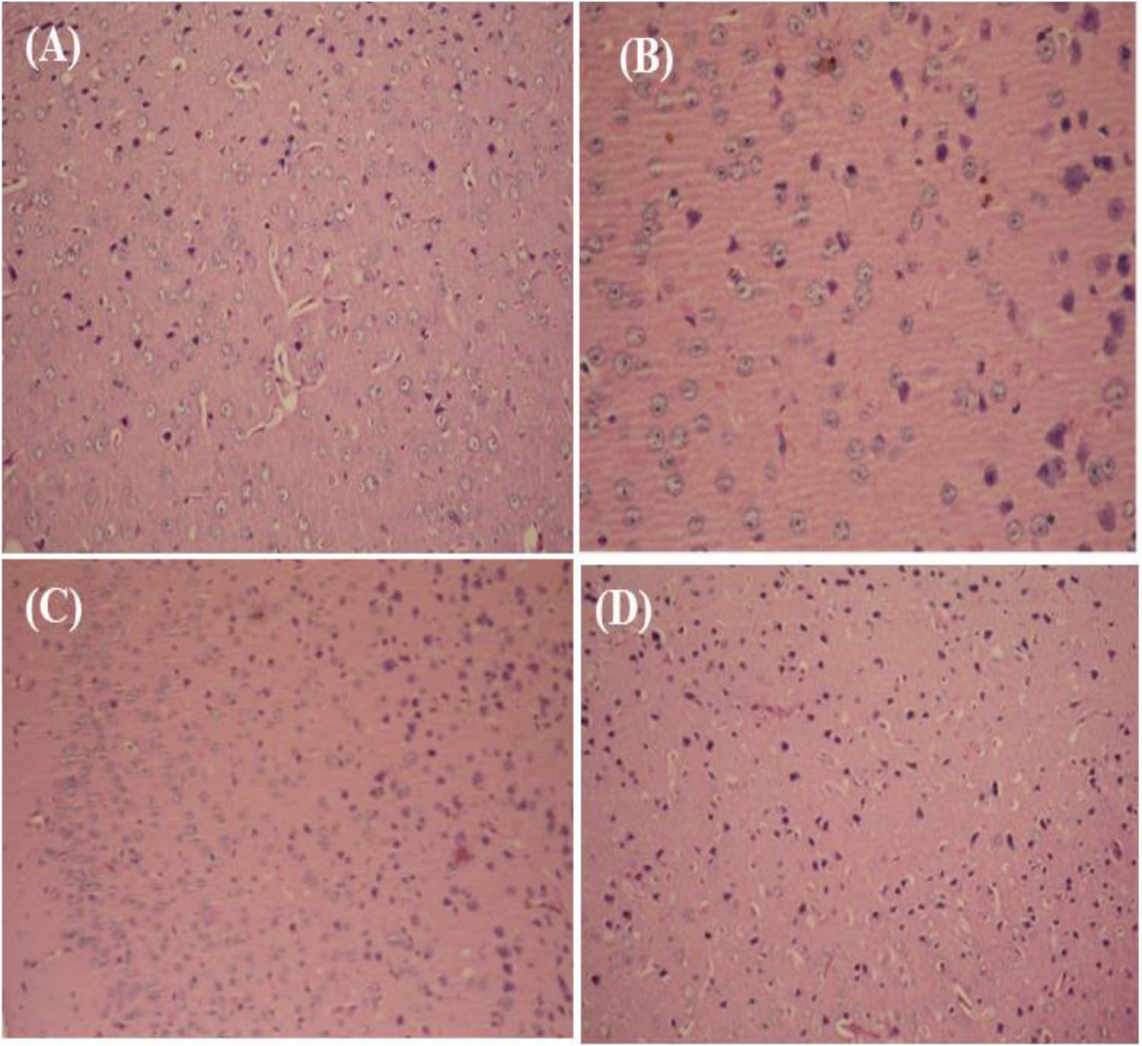
Histopathological examination of brain tissues in **A** control group normal neuroglial cells arranged in several layers; **B** HAA; pyknotic changes and nuclear hyperchromasia **C** ISPE, and **D** Prot. ISPE showed normal brain parenchyma histological appearances

In the control of photomicrograph of mice, brain sections showed normal neuroglial cells arranged in several layers. At the same time, the present study indicated that the brain section in the induction group showed edema in the brain parenchyma, with pyknotic changes and nuclear hyperchromasia. Chromatolysis of nuclear material, gliosis, and pyknotic neurons in the cortex was observed. In contrast, ISPE and Prot. ISPE showed normal brain parenchyma histological appearances (Fig. 6).

6. In silico study
7. Molecular docking study

Virtual screening using molecular docking simulations by Autodock Vina against 12 polyphenolic compounds identified using LCMS-MS from the marine sponge and downloaded from ZINC and PubChem databases, in addition to environmentally polluted samples (aromatic hydrocarbons) was performed *versus* aryl hydrocarbon receptor as a target. Preprocessing stage before docking, including preparing the target protein with native ligand CIT (citric acid) from PDB (5V0L), was done. The results of the docking experiment confirmed the in silico ligand–protein interaction. The docking experiment’s findings corroborated the predicted ligand–protein interaction. Important interactions between the natural products and the aryl hydrocarbon receptor were uncovered by molecular docking. Natural products could attach to the aryl hydrocarbon receptor’s active sites because a native inhibitor (CIT) fits into the crystal structure. Based on the active site study results, we know that certain amino acid residues are crucial for ligand binding: ASN B:61, LYS B:65, LEU B:49, ASP B:40, ASN B:43, ASP B:47, ASN B:43, LYS B:65, ALA B:50, LEU B:63, LEU B;46, ASN B:61, ALA B:50, ASP B:40, ALA B:50, SERB:51, ARG B:48, SER B:51, GLN B:57, THR B:44, VAL B:68, LEU B:46. Results of molecular docking illustrated in Table 5 revealed that all of the tested compounds exerted certain binding with the receptor where Rutin, ellagic acid, chlorogenic acid naringenin, and quercitrin showed the best fitting with Δ*G* values equal to − 7.6, − 7.3, − 7, − 6.5, and − 6.5 kcal/mol^−1^, respectively. The binding of the identified sponge natural components in the active pocket of AhR was illustrated in Figure S3.

**Table 5:** The results of docking interaction in terms of binding affinity (kcal/mol) with the aryl hydrocarbon receptor (PDB ID:5V0L) for natural products identified from the marine sponge and some PAHs such as anthracene, chrysene, pyrene, phenanthrene, and fluorene

Compound name	Database (Zinc/PubChem)	Affinity (Kcal/mol)	Amino acids residue
Chlorogenic acid (1)	ZINC6482465	** − 7.0**	PRO255, ALA227, CYS170, GLN144
Gallic acid (2)	ZINC1504	− 4.8	MET66, TRP67, GLU68, SER152, HIS265
Caffeic acid (3)	ZINC58172	− 5.1	MET66, SER152, LEU178, ILE181, THR200, HIS265
Rutin (4)	ZINC195062920	** − 7.6**	ASP115, THR123, ARG164, GLU205, ASN206, LYS210
*o*-Coumaric acid (5)	ZINC895911	− 5.1	ALA227, TRP228
Naringenin (6)	CID:932	** − 6.5**	MET66,SER152,LEU178,HIS265
Quercetin (7)	CID: 5,280,343	** − 6.6**	MET66, GLU68, SER152, HIS265, ASP266
Ellagic (8)	CID:5,281,855	** − 6.8**	MET66, MET153, PHE237, HIS265
Dihydroxybenzoic acid (9)	CID:72	− 4.7	LEU30,PHE31,GLU128
Methyl gallate (10)	CID:7428	− 4.7	GLN163,ARG164,SER166,ARG219,ASP222
Ferulic acid (11)	ZINC58258	− 5.4	MET66, LEU178, THR200, HIS265
Syringic acid (12)	CID: 10,742	− 4.5	ALA227,TRP228,GLN257,PHE280
Anthracene	ZINC1586329	− 6.2	LEU30, TYR132, LYS135
Chrysene	ZINC1693315	− 6.9	ALA227, PRO255, ILE284
Pyrene	ZINC1758808	− 6.4	ILE243, TYR246, PHE258, VAL260
Phenanthrene	ZINC967819	− 6.0	ILE243, PHE258, VAL260
Fluorene	ZINC968333	− 5.9	LEU30, PRO131, TYR132, LYS135

**Fig. 7:**
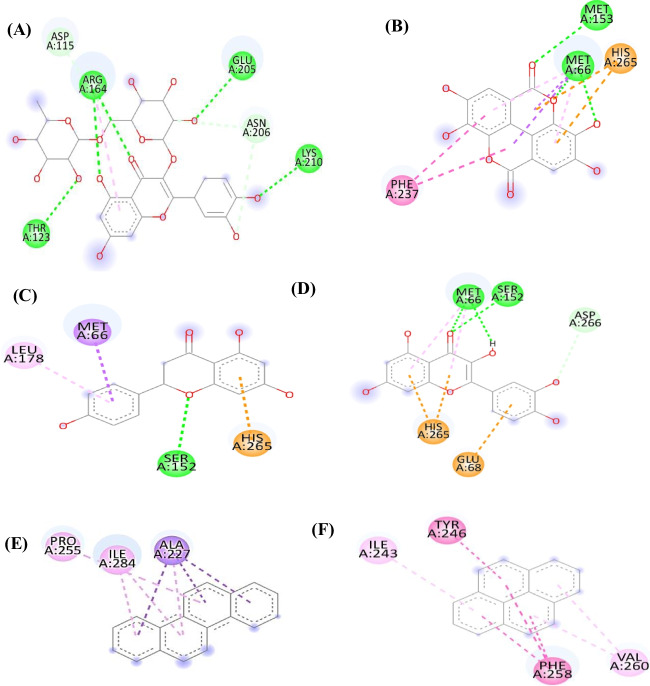
2D binding mode of rutin (**A**), ellagic acid (**B**), naringenin (**C**), quercetin (**D**), chrysene (**E**), and pyrene (**F**) within the active sites of aryl hydrocarbon receptor

2D binding mode of rutin declared the formation of many firm interactions with the receptor binding sites represented by four H-bonds with ARG164, GLU205, LYS210, and THR123 (Fig. 7). Meanwhile, naringenin binds with the AhR with an affinity of − 6.5 kcal/mole by conventional hydrogen bond, Pi- cation, π-δ, π- π shaped π-alkyl establishing interactions with different amino acid residues existing at the binding sites, which are MET66, SER152, LEU178, and HIS265. Besides, quercetin showed a distinguished interaction with the aryl hydrocarbon receptor accompanied by an affinity of − 6.6 kcal/mol displaying π-cation, π-alkyl, and hydrogen bond interactions, specifically MET66, GLU68, SER152, HIS265, and ASP266 amino acid moieties at the active site (Fig. 7). In addition, ellagic acid (8) firmly binds with the AhR with an affinity of − 6.8 kcal/mol, evidenced by the formation of multiple bonds with the amino acid residues MET66, MET153, PHE237, and HIS265 at the receptor active site. Molecular interaction profiles showed that polyphenolics form probable hydrogen bonds and atomic π-interactions. This greatly impacted the AhR-signaling pathway through their good inhibitory role in activating AhR-induced toxicological effects by some environmental pollution compounds such as aromatic hydrocarbon (Su et al. 2011).

(b) Pharmacodynamics, drug-likeness, and medicinal chemistry of ligands

In the current study, 12 polyphenolics were identified from *Ircinia* sp. sponge extract and subjected to ADME screening approaches to determine their pharmacodynamic and pharmacokinetic potentialities. *The Swiss performed *in silico* ADME analysi*s using predicted ADME parameters comprising pharmacokinetic properties, a drug-like nature, and medicinal chemistry. Swiss ADME is used to analyze the drug profiles and examine the safety of the promising candidates (DeLano 2002). Previous studies showed that polyphenolic compounds are powerful candidates against many diseases and can act as an antagonist and/or agonists for several target proteins to recover and ameliorate different diseases according to their dose and duration of treatment (Cotas et al. 2020). Results illustrated in Table S1 displayed the medicinal chemistry, lead-likeness, and pharmacokinetics properties of 12 polyphenolic compounds. It revealed that only rutin exhibited 3 violations with good ADME properties without toxicity in contrast to environmentally polluted samples (aromatic hydrocarbons), which showed high toxicity as seen in table S2 in the supplementary material.

## Discussion

Polycyclic aromatic hydrocarbons (PAHs) are carbon-containing chemical substances that persist in the environment and bioaccumulate through the food web, causing adverse effects on human health and the environment (Duran and Cravo-Laureau 2016). Herein, a variety of PAHs were used for the induction of kidney and brain toxicity. Several environmental contaminants comprise polycyclic aromatic hydrocarbons, in addition to naturally occurring compounds and endogenous ligands, which trigger the stimulation of AhR that consequently binds to its active sites leading to its dimerization with ARNT. This, in turn, results in the transcriptional activation of many metabolizing enzymes incorporated in xenobiotic phase I and phase II metabolism. Hence, the toxic effect of PAHs and related compounds can be explained by virtue of their activation by AhR (Dietrich 2016). Oxidative stress can eventually result from the stimulation of AhR via the metabolism of many ligands, such as PAHs and CYP1 enzyme induction. This can be achieved via the redox cycling of B[a]P-metabolite B[a]P-7,8-dihydrodiol where B[a]P-7,8-dihydrodiol is metabolized by aldo–keto reductases to B[a]P-7,8-diol. Various catechol groups concomitantly oxidize this by forming semiquinone radical and B[a]P-7,8-dione. Followed by its reduction by NADPH-mediated mechanisms to B[a]P-7,8-diol. Thus, this redox cycling results in the overproduction of superoxide anions and H_2_O_2_ and consequently in oxidative DNA damage (Park et al. 2009). However, it is worth highlighting that chemoprotective phytochemicals have multiple activities and may interact with several cellular receptors, including AhR. The AhR agonist/antagonist activity of the identified flavonoids in this study, such as kaempferol, naringenin, ellagic acid, quercetin, and myricetin, was investigated. Twelve polyphenolic compounds (Table 4) were inactive as agonists in the in silico study and were analyzed for AhR antagonist activities (Goya-Jorge et al. 2021).

Several studies showed that compounds such as PAHs could induce the induction of CYP1A with very different properties and origins (Nowicka-Bauer and Nixon 2020). The elevation level of kinases such as protein tyrosine kinase is reported through the phosphorylation of CYPs, kinases to regulate the AhR signal transduction pathway. This agrees with the current results as it has been tempting to suggest that PTKs might participate in the positive regulation of CYP1A because PTK inhibitors decrease CYP1A expression. This agrees with our results, as the level of PTK increases during PAH induction and reduces significantly after polyphenolic sponge extract administration. The polyphenolic sponge extract acts as a potent PTK inhibitor for PTK and affects either the activity of CYP1A or the level of CYP1A mRNA in the liver of the rats treated with benzo(a)pyrene (BP) (Babushkina et al. 2017).

Serum amyloid-A (SAA) and C-reactive protein (CRP) blood levels of acute-phase proteins are known to be biomarkers for several diseases by enhancing the production of foam cells and elevating the synthesis of plaques in the aorta (Hadrup et al. 2020; Legger et al. 2022). In mice, the dose-dependent acute pulmonary phase response is induced by airway exposure to insoluble particles, a complex systemic reaction characterized by changes in blood levels of SAA (Jeong et al. 2021). These data are thus consistent with current findings suggesting that SAA is associated with exposure to PAHs. Induction of prolonged acute phase response has been linked to inflammatory disease (Powell and Ghotbaddini 2014, Dimitrova-Shumkovska et al. 2023).

Several studies showed that phytochemicals activate the AhR weakly and act as AhR antagonists in one or more tests. These compounds include kaempferol, resveratrol, and different phytochemicals that block the transformation of cytosolic AhR induced by 2,3,7,8-tetrachlorodibenzo-p-dioxin (TCDD) in the rat liver. The phytochemicals that block TCDD-induced cytosolic AhR transformation in the rat liver include chrysin, kaempferol, quercetin, and other flavonoids (Goya-Jorge et al. 2021). Additionally, PAHs can cause kidney damage by causing inflammatory effects. Significant increases in creatinine and urea levels in PAHs treated rats were found in the current results, confirming the mice’s renal dysfunction. Elevated urea indicates diminished renal epithelium reabsorption, whereas high serum creatinine revealed impairment in renal function, mainly in rats treated with PAHs for glomerular filtration rate.

Additionally, the PAHs increase the serum levels of kidney functional parameters, and this was significantly reversed by co-treatment with the sponge extract. In addition, the pattern of antioxidant enzyme activity in both kidney and brain tissues was enhanced. A number of factors play a role in this, including GSH and GSH-related enzymes, GST, GPx, GR, and the concentration of MDA. As part of the glutathione redox cycle, they convert H_2_O_2_ and lipid peroxides into non-toxic products. The level of MDA in cells and tissues is a well-established indicator of oxidative stress, a biomarker of lipid peroxidation (Meli et al. 2020). According to the current study, mice treated with PAHs showed marked reductions in renal GSH. In the kidneys of mice exposed to PAHs, the antioxidant defense system was impaired, inflammation was observed, and oxidative stress was observed. The findings are consistent with prior reports that PAH exposure increases oxidative stress and inflammation in PAH-treated mice (Chepelev et al. 2016). Furthermore, co-treatment with sponge extract markedly improved the status of antioxidants and enhanced renal histology, thus revealing sponge extract’s potent protective role in mice treated with PAHs.

In addition, there are presumably many mechanisms through which PAHs produce neurotoxicity. To begin, PAHs may change the gene expression of the *N*-methyl-d-aspartate receptor, a glutamate receptor, and ion channel protein in nerve cells. As one of the best-studied transcription factors, the AhR plays a role in synaptic plasticity and memory (Mortamais et al. 2017; Nowicka-Bauer and Nixon 2020). Furthermore, PAHs can cause oxidative stress associated with alterations in gene expression, cellular signaling, membrane integrity, neurotransmission, and even neuronal death (Li et al. 2020). Results illustrated in the current study showed that PAHs significantly increase the oxidative stress markers in the brain with a concomitant reduction in the antioxidant biomarkers.

On the contrary, co-treatment with sponge extract significantly decreased the oxidative stress markers in the brain with concomitant elevation in the antioxidant biomarkers and amelioration of brain histology. Virtual screening using molecular docking simulations of 12 polyphenolic compounds identified from the marine sponge was performed *against* the aryl hydrocarbon receptor as a target. The results of the docking experiment confirmed the in silico ligand–protein interaction. They revealed principal interactions that are transpiring between the natural products and the aryl hydrocarbon receptor where all tested compounds exerted certain binding with the receptor where rutin, ellagic acid, chlorogenic acid kaempferol, and quercetin showed the best fitting and thus confirming our in vitro and in vivo studies. Besides, polyphenolics identified from *Ircinia* sp. sponge extract were subjected to ADME screening approaches to determine their pharmacodynamic and pharmacokinetic potentialities. Results revealed that only rutin exhibited three violations with good ADME properties without toxicity in contrast to environmentally polluted samples (Aromatic Hydrocarbons), which showed high toxicity.

We want to brief and highlight the main important finding in our study. The ISPE extract is the first to be investigated in an in vivo study as both neuroprotective and nephroprotective drug candidates. The effect of ISPE was obviously seen in improving kidney function, antioxidants and diminished all inflammatory (IL6, TNF), neurodegenerative biomarkers (SAA), and oxidative stress. The study used different screening phases in vitro, in vivo, and computational drug design approaches. All these results were confirmed with histopathological findings. This study has some limitations, which we can summarize in a further investigation that should be carried out to purify the pure compounds or study the effect of ISPE compounds individually.

## Conclusions

Thus, it can be concluded that ISPE revealed promising neuroprotective and nephroprotective effects manifested by several approaches (in vitro, in vivo, and computational studies) and further consolidated by computational approaches. ISPE showed pronounced antioxidant and anti-cholinesterase activity in vitro. Besides, it significantly ameliorated neurotoxicity and nephrotoxicity triggered by polycyclic aromatic hydrocarbon (PAH) toxicity in vivo. It revealed a reduction in serum urea, uric acid, and creatinine. It also revealed a decline in MDA and total proteins (TP) in kidney and brain tissues with concomitant elevation in GSH and GST. It also showed a reduction in the inflammatory and pre-cancerous biomarkers (SAA and PTK). The findings of both kidney and brain tissue histopathological examinations support this conclusion. ISPE polyphenolic content as revealed by LC–MS-MS could contribute to these protective effects, which were subsequently confirmed by molecular docking at the active sites of aryl hydrocarbon receptors. Consequently, ISPE showed promising neuroprotective properties and nephroprotective against PAH toxicity that preclinical studies should follow to confirm the obtained results.

## Supplementary Information

Below is the link to the electronic supplementary material.

Supplementary file1 (DOCX 1.48 MB)

Supplementary file2 (DOCX 129 KB)

## Data Availability

Data are available in the manuscript.
