# Correction to: Neuroprotective and nephroprotective effects of *Ircinia* sponge in polycyclic aromatic hydrocarbons (PAHs) induced toxicity in animal model: a pharmacological and computational approach

**DOI:** 10.1007/s11356-023-28425-9

**Published:** 2023-06-27

**Authors:** Asmaa Nabil-Adam, Fadia S. Youssef, Mohamed L. Ashour, Mohamed A. Shreadah

**Affiliations:** 1 Marine Biotechnology and Natural Products Lab (MBNP), National Institute of Oceanography & Fisheries (NIOF), Alexandria, Egypt; 2 Department of Pharmacognosy, Faculty of Pharmacy, Ain-Shams University, Abbasia, Cairo, 11566 Egypt; 3 Department of Pharmaceutical Sciences, Pharmacy Program, Batterjee Medical College, PO Box 6231, Jeddah, 21442 Saudi Arabia

**Correction to: Environmental Science and Pollution Research**

10.1007/s11356-023-27916-z

The supplementary materials contains the Reviewers comments.

The Original article has been corrected.

