# Supplementary material for: Neuroprotective and nephroprotective effects of Ircinia sponge in polycyclic aromatic hydrocarbons (PAHs) induced toxicity in animal model: a pharmacological and computational approach
Source: Environ Sci Pollut Res Int. 2023 Jun 15;30(34):82162–77. doi: 10.1007/s11356-023-27916-z (PMC10349714; doi:10.1007/s11356-023-27916-z)
Supplement: Supplementary file 1 — Supplementary file1 (DOCX 1.48 MB) [file 11356_2023_27916_MOESM1_ESM.docx]

**Neuroprotective and Nephroprotective Effects of *Ircinia sponge* in Polycyclic Aromatic Hydrocarbons (PAHs) induced toxicity in animal model: A pharmacological and computational approach**

**Supplementary material**

**Figure S1** LC/MS/MS chromatogram of *Ircinia sp.* sponge extract showing the different distribution of polyphenolic compounds

**Figure S2** The heat map showing the distribution of polyphenolic compounds of the *Ircinia sp.* sponge extract

**Figure S3** The binding of the identified sponge natural components in the active pocket of AhR

Figure S4 : The Experimental design

**Table S1** The pharmacokinetics, medicinal chemistry and drug-likeness for each compound isolated from marine natural products

**Table S2** The pharmacokinetics, medicinal chemistry and drug likeness for each compound isolated from environmental polluted samples (aromatic hydrocarbons).

**LC/MS/MS Chromatograme**

- **Standard (100ppb) :**


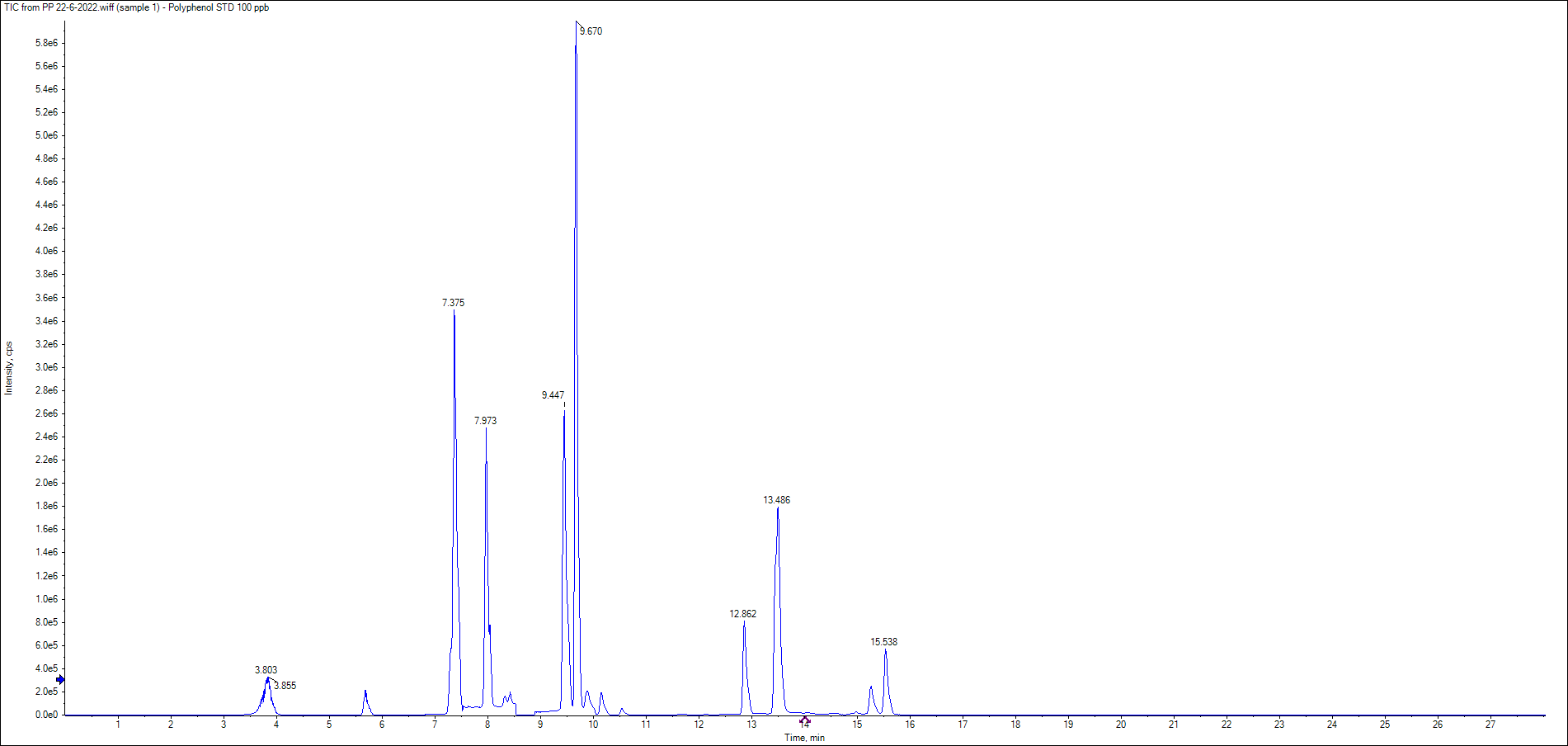


- *Ircinia sp.* sponge**:**


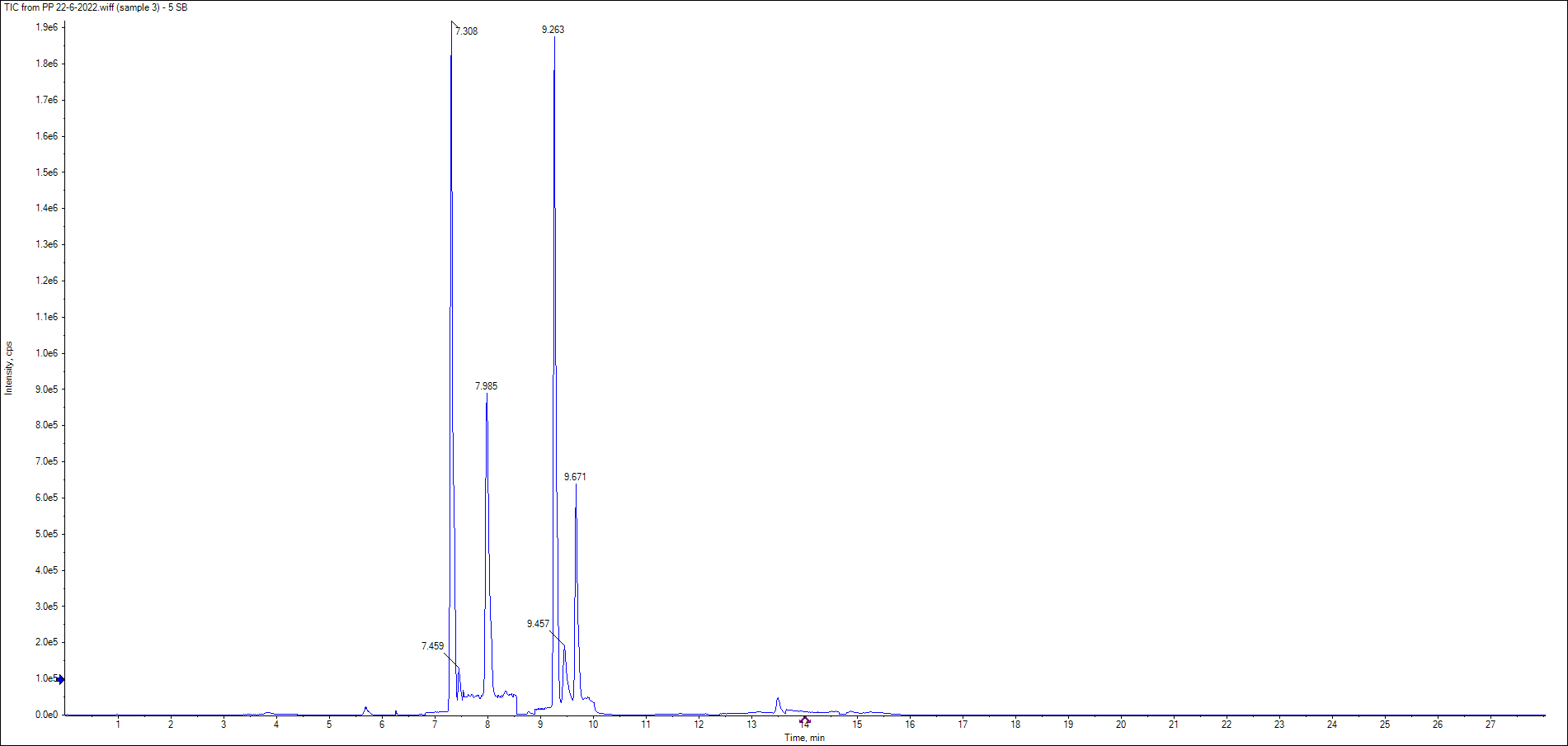


**Figure S1** LC/MS/MS chromatogram of *Ircinia sp.* sponge extract showing the different distribution of polyphenolic compounds


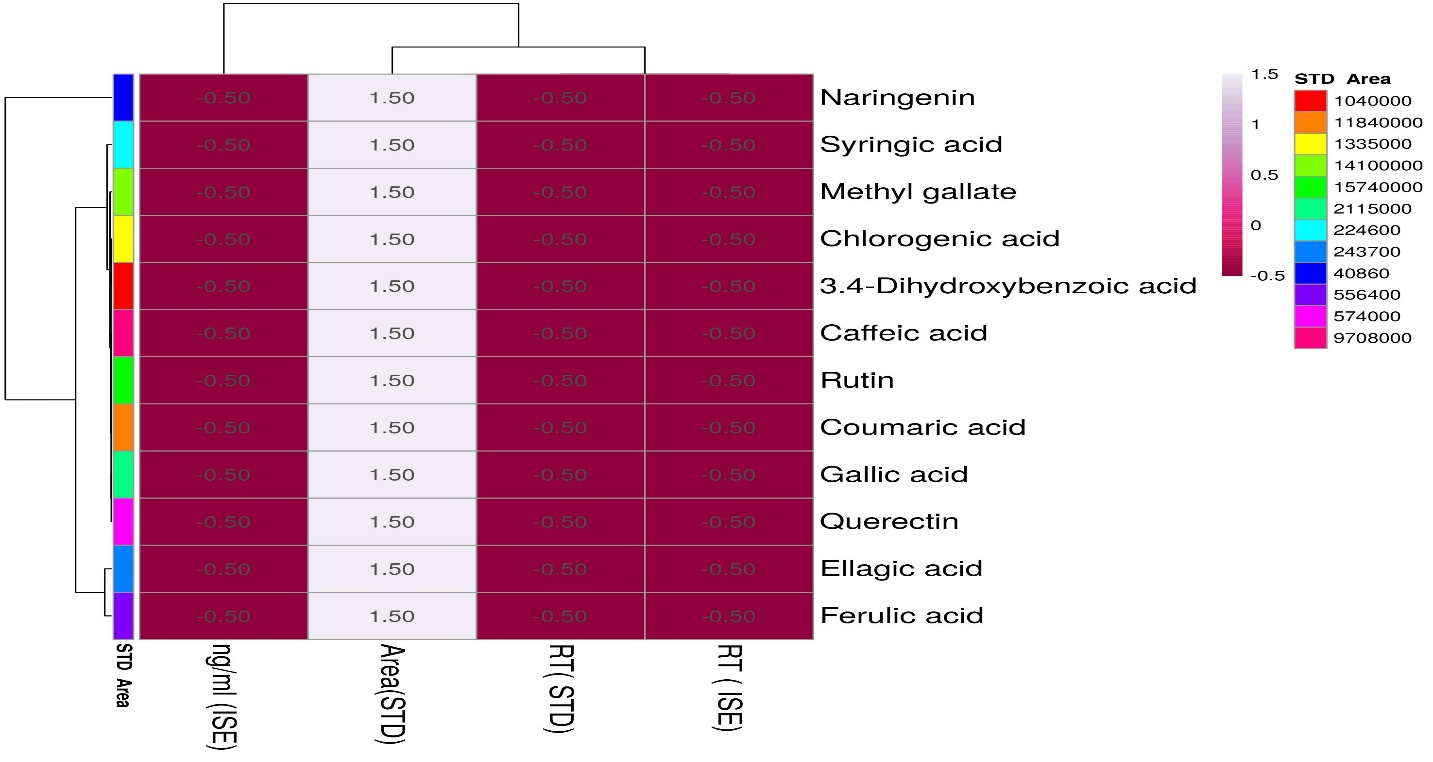


**Figure S2** The heat map showing the distribution of polyphenolic compounds of the *Ircinia sp.* sponge extract

**Figure S3** The binding of the identified sponge natural components in the active pocket of AhR

| **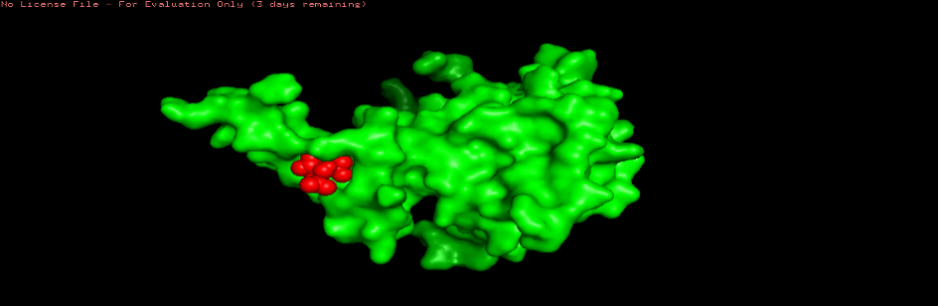** | **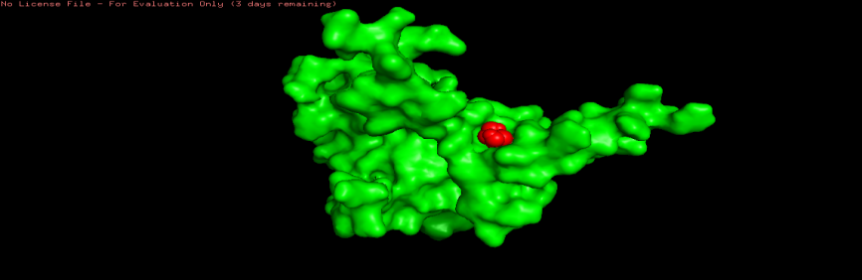** | **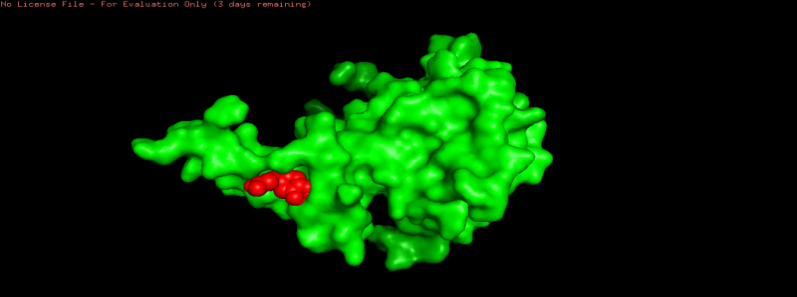** | **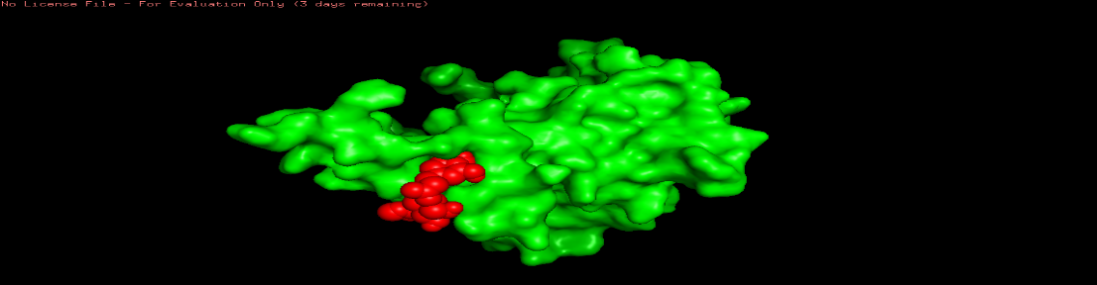** |
| --- | --- | --- | --- |
| **CIT (citric acid)** | **Glycerol (GOT)** | **Caffeic Acid** | **Chrysene** |
| 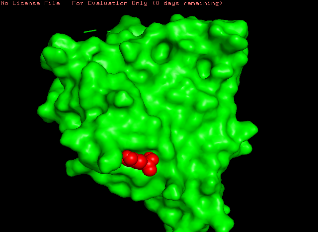 | **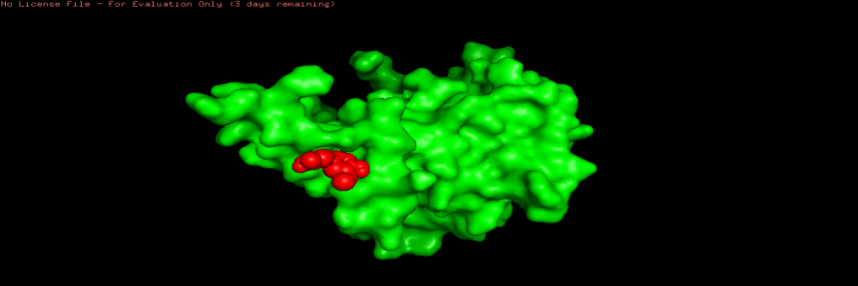** | **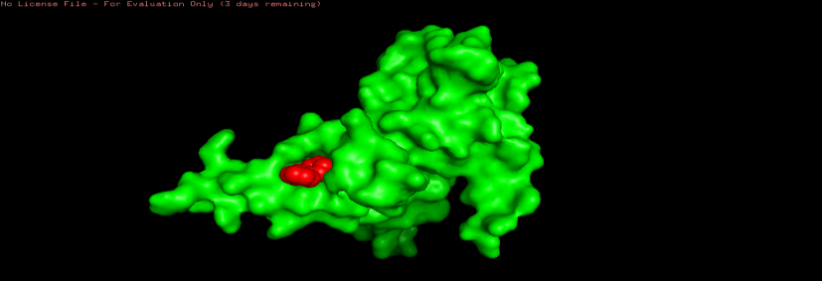** | **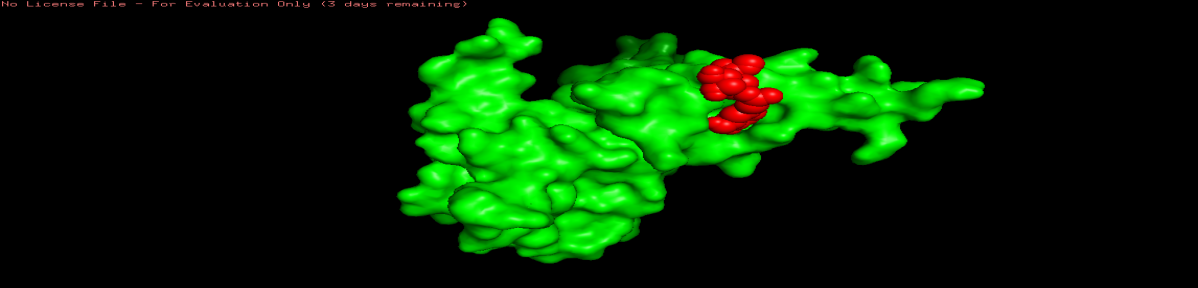** |
| **3.4-Dihydroxybenzoic acid** | **Ferulate** | **Phenanthrene** | **Chlorogenic Acid** |
| **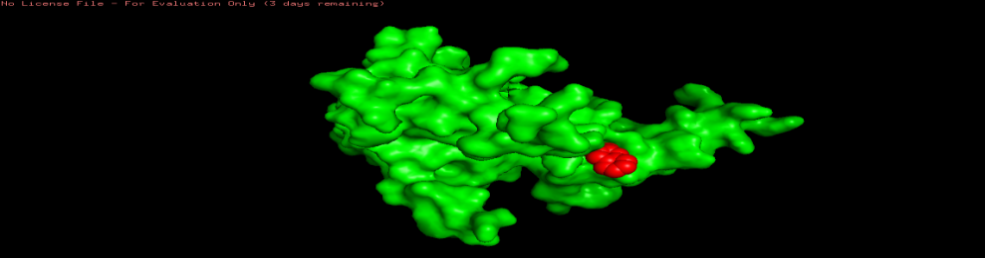** | 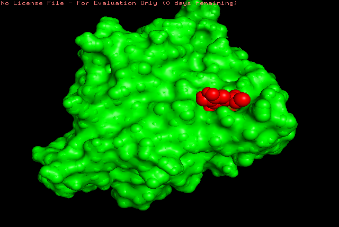 | **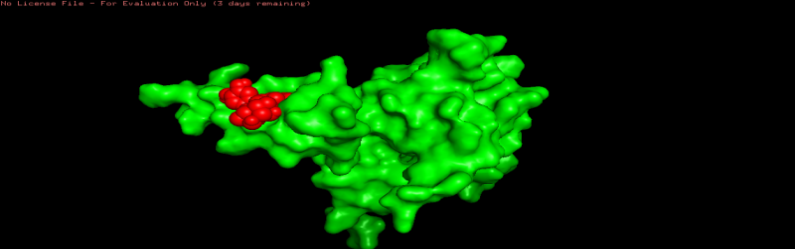** | 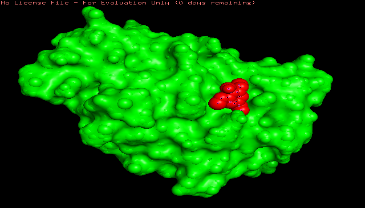 |
| **Anthracene** | **Methyl gallate** | **Quercetin** | **Syringic acid** |
| **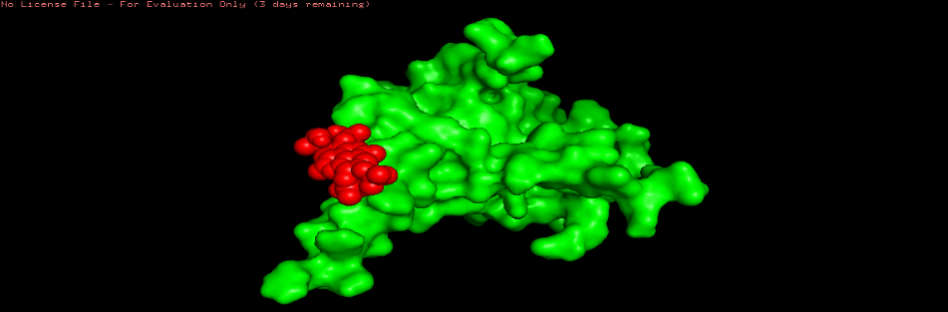** | **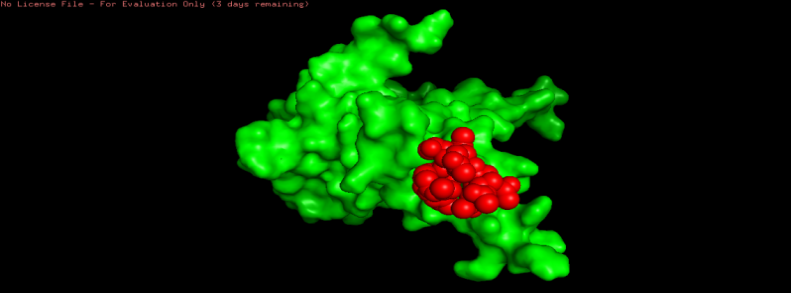** | **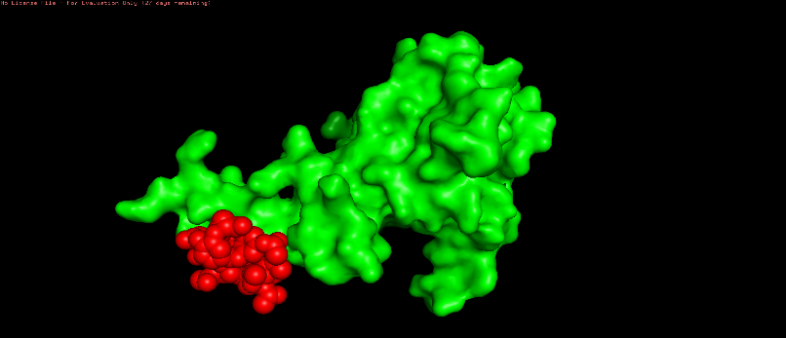** | **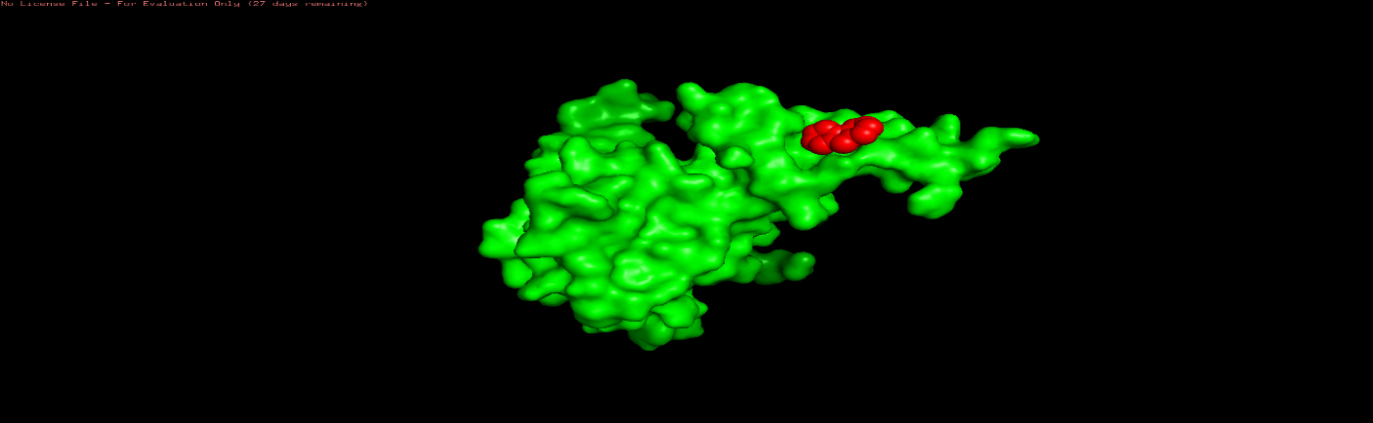** |
| **Ellagic Acid** | **Pyrene** | **Rutin** | **Fluorene** |

Figure (4) : The diagram represents the experimental design of the *In-vivo* study

**Table S1** The pharmacokinetics, medicinal chemistry, and drug-likeness for each compound isolated from marine natural products.

|  | **Pharmacokinetics** | **Medicinal Chemistry** | **Drug likeness** |
| --- | --- | --- | --- |
| Syringic acid | GI absorption High  BBB permeant No  P-gp substrate No  CYP1A2 inhibitor No  CYP2C19 inhibitor No  CYP2C9 inhibitor No  CYP2D6 inhibitor No  CYP3A4 inhibitor No  Log Kp (skin permeation) -6.77 cm/s | PAINS 0 alert  Brenk 0 alert  Leadlikeness No; 1 violation: MW<250  Synthetic accessibility 1.70 | Lipinski Yes; 0 violation  Ghose Yes  Veber Yes  Egan Yes  Muegge No; 1 violation: MW<200  Bioavailability Score 0.56 |
| **Caffeic Acid** | GI absorption High  BBB permeant No  P-gp substrate No  CYP1A2 inhibitor No  CYP2C19 inhibitor No  CYP2C9 inhibitor No  CYP2D6 inhibitor No  CYP3A4 inhibitor No  Log Kp (skin permeation) -6.58 cm/s | PAINS 1 alert: catechol_A  Brenk 2 alerts: catechol, michael_acceptor_1  Lead likeness No; 1 violation: MW<250  Synthetic accessibility 1.81 | Lipinski Yes; 0 violation  Ghose Yes  Veber Yes  Egan Yes  Muegge No; 1 violation: MW<200  Bioavailability Score 0.56 |
| **Ferulate** | GI absorption High  BBB permeant Yes  P-gp substrate No  CYP1A2 inhibitor No  CYP2C19 inhibitor No  CYP2C9 inhibitor No  CYP2D6 inhibitor No  CYP3A4 inhibitor No  Log Kp (skin permeation) -6.41 cm/s | PAINS 0 alert  Brenk 1 alert: michael_acceptor_1  Leadlikeness No; 1 violation: MW<250  Synthetic accessibility 1.93 | Lipinski Yes; 0 violation  Ghose Yes  Veber Yes  Egan Yes  Muegge No; 1 violation: MW<200  Bioavailability Score 0.85 |
| **Methyl gallate** | GI absorption High  BBB permeant No  P-gp substrate No  CYP1A2 inhibitor No  CYP2C19 inhibitor No  CYP2C9 inhibitor No  CYP2D6 inhibitor No  CYP3A4 inhibitor No  Log Kp (skin permeation) -6.81 cm/s | PAINS 1 alert: catechol_A  Brenk 1 alert: catechol  Leadlikeness No; 1 violation: MW<250  Synthetic accessibility 1.50 | Lipinski Yes; 0 violation  Ghose Yes  Veber Yes  Egan Yes  Muegge No; 1 violation: MW<200  Bioavailability Score 0.55 |
| **Quercetin** | GI absorption High  BBB permeant No  P-gp substrate No  CYP1A2 inhibitor Yes  CYP2C19 inhibitor No  CYP2C9 inhibitor No  CYP2D6 inhibitor Yes  CYP3A4 inhibitor Yes  Log Kp (skin permeation) -7.05 cm/s | PAINS 1 alert: catechol_A  Brenk 1 alert: catechol  Leadlikeness Yes  Synthetic accessibility 3.23 | Lipinski Yes; 0 violation  Ghose Yes  Veber Yes  Egan Yes  Muegge Yes  Bioavailability Score 0.55 |
| **Chlorogenic Acid** | GI absorption Low  BBB permeant No  P-gp substrate No  CYP1A2 inhibitor No  CYP2C19 inhibitor No  CYP2C9 inhibitor No  CYP2D6 inhibitor No  CYP3A4 inhibitor No  Log Kp (skin permeation) -8.62 cm/s | PAINS 1 alert: catechol_A  Brenk 3 alerts: catechol, michael_acceptor_1, more_than_2_esters  Leadlikeness No; 1 violation: MW>350  Synthetic accessibility 4.27 | Lipinski Yes; 0 violation  Ghose No; 1 violation: WLOGP<-0.4  Veber No; 1 violation: TPSA>140  Egan No; 1 violation: TPSA>131.6  Muegge No; 1 violation: TPSA>150  Bioavailability Score 0.55 |
| **Naringenin** | GI absorption High  BBB permeant No  P-gp substrate Yes  CYP1A2 inhibitor Yes  CYP2C19 inhibitor No  CYP2C9 inhibitor No  CYP2D6 inhibitor No  CYP3A4 inhibitor Yes  Log Kp (skin permeation) -6.17 cm/s | PAINS 0 alert  Brenk 0 alert  Leadlikeness Yes  Synthetic accessibility 3.01 | Lipinski Yes; 0 violation  Ghose Yes  Veber Yes  Egan Yes  Muegge Yes  Bioavailability Score 0.55 |
| **Ellagic Acid** | GI absorption High  BBB permeant No  P-gp substrate No  CYP1A2 inhibitor Yes  CYP2C19 inhibitor No  CYP2C9 inhibitor No  CYP2D6 inhibitor No  CYP3A4 inhibitor No  Log Kp (skin permeation) -7.36 cm/s | PAINS 1 alert: catechol_A  Brenk 3 alerts: catechol, cumarine, polycyclic_aromatic_hydrocarbon_3  Leadlikeness Yes  Synthetic accessibility 3.17 | Lipinski Yes; 0 violation  Ghose Yes  Veber No; 1 violation: TPSA>140  Egan No; 1 violation: TPSA>131.6  Muegge Yes  Bioavailability Score 0.55 |
| **O-Coumarate** | GI absorption High  BBB permeant Yes  P-gp substrate No  CYP1A2 inhibitor No  CYP2C19 inhibitor No  CYP2C9 inhibitor No  CYP2D6 inhibitor No  CYP3A4 inhibitor No  Log Kp (skin permeation) -6.26 cm/s | PAINS 0 alert  Brenk 1 alert: michael_acceptor_1  Leadlikeness No; 1 violation: MW<250  Synthetic accessibility 1.61 | Lipinski Yes; 0 violation  Ghose Yes  Veber Yes  Egan Yes  Muegge No; 1 violation: MW<200  Bioavailability Score 0.85 |
| **3,4Dihydroxybenzoic acid** | GI absorption High  BBB permeant No  P-gp substrate No  CYP1A2 inhibitor No  CYP2C19 inhibitor No  CYP2C9 inhibitor No  CYP2D6 inhibitor No  CYP3A4 inhibitor Yes  Log Kp (skin permeation) -6.42 cm/s | PAINS 1 alert: catechol_A  Brenk 1 alert: catechol  Leadlikeness No; 1 violation: MW<250  Synthetic accessibility 1.07 | Lipinski Yes; 0 violation  Ghose No; 3 violations: MW<160, MR<40, #atoms<20  Veber Yes  Egan Yes  Muegge No; 1 violation: MW<200  Bioavailability Score 0.56 |
| **Gallic acid** | GI absorption High  BBB permeant No  P-gp substrate No  CYP1A2 inhibitor No  CYP2C19 inhibitor No  CYP2C9 inhibitor No  CYP2D6 inhibitor No  CYP3A4 inhibitor Yes  Log Kp (skin permeation) -6.84 cm/s | PAINS 1 alert: catechol_A  Brenk 1 alert: catechol  Leadlikeness No; 1 violation: MW<250  Synthetic accessibility 1.22 | Lipinski Yes; 0 violation  Ghose No; 2 violations: MR<40, #atoms<20  Veber Yes  Egan Yes  Muegge No; 1 violation: MW<200  Bioavailability Score 0.56 |
| **Rutin** | GI absorption Low  BBB permeant No  P-gp substrate Yes  CYP1A2 inhibitor No  CYP2C19 inhibitor No  CYP2C9 inhibitor No  CYP2D6 inhibitor No  CYP3A4 inhibitor No  Log Kp (skin permeation) -10.26 cm/s | PAINS 1 alert: catechol_A  Brenk 1 alert: catechol  Leadlikeness No; 1 violation: MW>350  Synthetic accessibility 6.52 | Lipinski No; 3 violations: MW>500, NorO>10, NHorOH>5  Ghose No; 4 violations: MW>480, WLOGP<-0.4, MR>130, #atoms>70  Veber No; 1 violation: TPSA>140  Egan No; 1 violation: TPSA>131.6  Muegge No; 4 violations: MW>600, TPSA>150, H-acc>10, H-don>5  Bioavailability Score 0.17 |

**Table S2** The pharmacokinetics, medicinal chemistry and drug likeness for each compound isolated from environmental polluted samples (aromatic hydrocarbons)

|  | **Pharmacokinetics** | **Medicinal Chemistry** | **Drug likeness** |
| --- | --- | --- | --- |
| **Phenanthrene** | GI absorption Low  BBB permeant Yes  P-gp substrate No  CYP1A2 inhibitor Yes  CYP2C19 inhibitor Yes  CYP2C9 inhibitor No  CYP2D6 inhibitor No  CYP3A4 inhibitor No  Log Kp (skin permeation) -4.22 cm/s | PAINS 0 alert  Brenk 1 alert: polycyclic_aromatic_hydrocarbon_3  Leadlikeness No; 2 violations: MW<250, XLOGP3>3.5  Synthetic accessibility 1.00 | Lipinski Yes; 1 violation: MLOGP>4.15  Ghose Yes  Veber Yes  Egan Yes  Muegge No; 2 violations: MW<200, Heteroatoms<2  Bioavailability Score 0.55 |
| **Anthracene** | GI absorption Low  BBB permeant Yes  P-gp substrate No  CYP1A2 inhibitor Yes  CYP2C19 inhibitor Yes  CYP2C9 inhibitor No  CYP2D6 inhibitor No  CYP3A4 inhibitor No  Log Kp (skin permeation) -4.23 cm/s | PAINS 0 alert  Brenk 1 alert: polycyclic_aromatic_hydrocarbon_2  Leadlikeness No; 2 violations: MW<250, XLOGP3>3.5  Synthetic accessibility 1.00 | Lipinski Yes; 1 violation: MLOGP>4.15  Ghose Yes  Veber Yes  Egan Yes  Muegge No; 2 violations: MW<200, Heteroatoms<2  Bioavailability Score 0.55 |
| **Pyrene** | GI absorption Low  BBB permeant No  P-gp substrate No  CYP1A2 inhibitor Yes  CYP2C19 inhibitor No  CYP2C9 inhibitor No  CYP2D6 inhibitor No  CYP3A4 inhibitor No  Log Kp (skin permeation) -4.07 cm/s | PAINS 0 alert  Brenk 1 alert: polycyclic_aromatic_hydrocarbon_3  Leadlikeness No; 2 violations: MW<250, XLOGP3>3.5  Synthetic accessibility 1.00 | Lipinski Yes; 1 violation: MLOGP>4.15  Ghose Yes  Veber Yes  Egan Yes  Muegge No; 1 violation: Heteroatoms<2  Bioavailability Score 0.55 |
| **Fluorene** | GI absorption Low  BBB permeant Yes  P-gp substrate Yes  CYP1A2 inhibitor Yes  CYP2C19 inhibitor Yes  CYP2C9 inhibitor No  CYP2D6 inhibitor No  CYP3A4 inhibitor No  Log Kp (skin permeation) -4.35 cm/s | PAINS 0 alert  Brenk 0 alert  Leadlikeness No; 2 violations: MW<250, XLOGP3>3.5  Synthetic accessibility 2.00 | Lipinski Yes; 1 violation: MLOGP>4.15  Ghose Yes  Veber Yes  Egan Yes  Muegge No; 2 violations: MW<200, Heteroatoms<2  Bioavailability Score 0.55 |
| **Chrysene** | GI absorption Low  BBB permeant No  P-gp substrate No  CYP1A2 inhibitor Yes  CYP2C19 inhibitor No  CYP2C9 inhibitor No  CYP2D6 inhibitor No  CYP3A4 inhibitor No  Log Kp (skin permeation) -3.57 cm/s | PAINS 0 alert  Brenk 1 alert: polycyclic_aromatic_hydrocarbon_3  Leadlikeness No; 2 violations: MW<250, XLOGP3>3.5 | Lipinski Yes; 1 violation: MLOGP>4.15  Ghose Yes  Veber Yes  Egan Yes  Muegge No; 2 violations: XLOGP3>5, Heteroatoms<2  Bioavailability Score 0.55 |

**List of abbreviations**

1. PAH Polycyclic aromatic hydrocarbons (PAHs)
2. **ISE *Ircinia sp*. Extract**
3. **ABTS 2,2'-azino-bis(3-ethylbenzothiazoline-6-sulfonic acid**
4. **DPPH 2,2-diphenyl-1-picryl-hydrazyl-hydrate**
5. **LPO lipid peroxidation**
6. **GSH, Glutathione**
7. **GST Glutathione S-transferases**
8. **PTK, Protein tyrosine kinases**
9. **AHR Aryl hydrocarbon receptor (AHR**
10. **SAA serum amyloid A**
11. **LC-MS-MS**
12. **ADME absorption, distribution, metabolism, and excretion)**
13. **ROS Reactive oxygen species**
14. **CNS central nervous system**
15. **CKD chronic kidney disease (CKD)**
16. **CVD cardiovascular disease (CVD)**
17. **GC-MS Gas chromatography–mass spectrometry**
18. **AChI acetylcholinesterase inhibitory**
19. **MDA malondialdehyde**
20. **ARNT. aryl hydrocarbon receptor nuclear translocator**
21. **GPx Glutathione Peroxidase**
22. **GR Glutathione reductase**
