# Supplementary figures and images for: Neuroprotective and nephroprotective effects of Ircinia sponge in polycyclic aromatic hydrocarbons (PAHs) induced toxicity in animal model: a pharmacological and computational approach
Source: Environ Sci Pollut Res Int. 2023 Jun 15;30(34):82162–77. doi: 10.1007/s11356-023-27916-z (PMC10349714; doi:10.1007/s11356-023-27916-z)

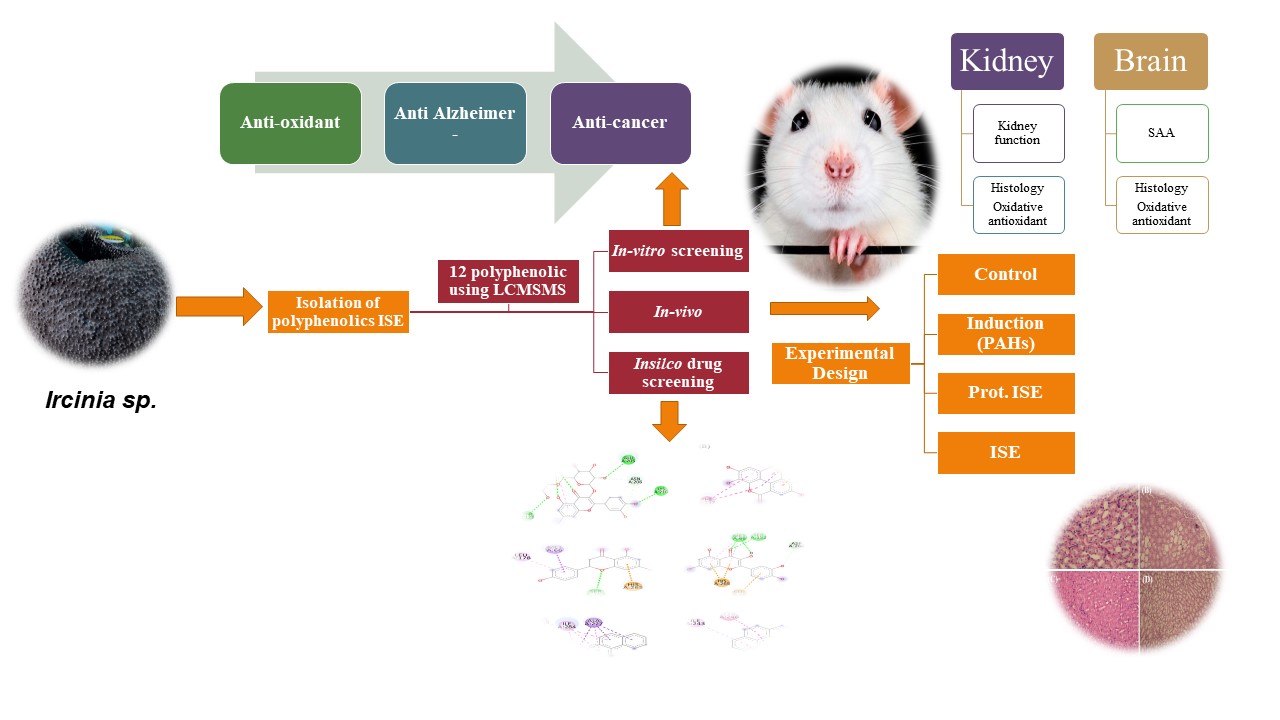


The outline of all the experiments

Supplement: Supplementary file 2 — Supplementary file2 (DOCX 129 KB) [file 11356_2023_27916_MOESM2_ESM.docx]
